# Dynamic Regulation of Auxin Response during Rice Development Revealed by Newly Established Hormone Biosensor Markers

**DOI:** 10.3389/fpls.2017.00256

**Published:** 2017-03-07

**Authors:** Jing Yang, Zheng Yuan, Qingcai Meng, Guoqiang Huang, Christophe Périn, Charlotte Bureau, Anne-Cécile Meunier, Mathieu Ingouff, Malcolm J. Bennett, Wanqi Liang, Dabing Zhang

**Affiliations:** ^1^Key Laboratory of Systems Biomedicine, Ministry of Education, Shanghai Center for Systems Biomedicine, Shanghai Jiao Tong UniversityShanghai, China; ^2^Joint International Research Laboratory of Metabolic & Developmental Sciences, Shanghai Jiao Tong University–University of Adelaide Joint Centre for Agriculture and Health, School of Life Sciences and Biotechnology, Shanghai Jiao Tong UniversityShanghai, China; ^3^CIRAD, UMR AGAPMontpellier, France; ^4^Centre for Plant Integrative Biology, School of Biosciences, University of NottinghamSutton Bonington, UK; ^5^School of Agriculture, Food and Wine, University of AdelaideUrrbrae, SA, Australia

**Keywords:** rice, auxin, reporter, lateral root formation, inflorescence, spikelet, meristem

## Abstract

The hormone auxin is critical for many plant developmental processes. Unlike the model eudicot plant *Arabidopsis* (*Arabidopsis thaliana*), auxin distribution and signaling in rice tissues has not been systematically investigated due to the absence of suitable auxin response reporters. In this study we observed the conservation of auxin signaling components between *Arabidopsis* and model monocot crop rice (*Oryza sativa*), and generated complementary types of auxin biosensor constructs, one derived from the Aux/IAA-based biosensor *DII-VENUS* but constitutively driven by maize ubiquitin-1 promoter, and the other termed *DR5*-VENUS in which a synthetic auxin-responsive promoter (*DR5*_*rev*_) was used to drive expression of the yellow fluorescent protein (YFP). Using the obtained transgenic lines, we observed that during the vegetative development, accumulation of *DR5*-VENUS signal was at young and mature leaves, tiller buds and stem base. Notably, abundant *DR5*-VENUS signals were observed in the cytoplasm of cortex cells surrounding lateral root primordia (LRP) in rice. In addition, auxin maxima and dynamic re-localization were seen at the initiation sites of inflorescence and spikelet primordia including branch meristems (BMs), female and male organs. The comparison of these observations among *Arabidopsis*, rice and maize suggests the unique role of auxin in regulating rice lateral root emergence and reproduction. Moreover, protein localization of auxin transporters PIN1 homologs and GFP tagged OsAUX1 overlapped with *DR5*-VENUS during spikelet development, helping validate these auxin response reporters are reliable markers in rice. This work firstly reveals the direct correspondence between auxin distribution and rice reproductive and root development at tissue and cellular level, and provides high-resolution auxin tools to probe fundamental developmental processes in rice and to establish links between auxin, development and agronomical traits like yield or root architecture.

## Introduction

The phytohormone auxin (indole-3-acetic acid, IAA) regulates many critical growth and developmental processes in plants. IAA is synthesized in subsets of plant cells and then actively transported from cell to cell through polar transport. Development of effective hormone biosensors to visualize auxin distribution *in vivo* is needed to dissect the functions of this key hormone during plant development. In *Arabidopsis*, the most widely applied tool is *DR5*-GFP which uses a synthetic auxin-responsive promoter (*DR5*_*rev*_) to drive the expression of green fluorescent protein (Heisler et al., 2005). Auxin can be detected using *DR5*-GFP transgenic lines, despite of its indirect connection with auxin abundance *in vivo*, and the slow time-scale of its auxin induced response (taking several hours from induction) which is not optimized to study fast biological processes such as tropic responses (Zhao et al., 2014). Brunoud et al. (2012) developed an alternative reporter system employing the *CaMV35S* promoter to constitutively drive expression of the DII-VENUS sequence in which the YFP VENUS reporter was fused to the auxin degron sequence called DII, present in Aux/IAA repressor proteins. The presence of auxin triggers the degradation of the DII-VENUS fusion protein, where the reduction in reporter fluorescence intensity is proportional to lAA levels in cells. Hence, subtle differences in auxin abundance can be visualized through changes in fluorescence, allowing high-resolution spatio-temporal changes in auxin distribution and response during plant growth and development (Brunoud et al., 2012). These two systems have been extensively used to characterize functions of genes associated with auxin signaling (Steenackers et al., 2016), gravitropic response (Band et al., 2012; Zou et al., 2016) and stomatal patterning (Le et al., 2014). New generations of DII-VENUS and *DR5*-GFP have also been recently developed. R2D2 integrates an auxin sensitive DII-VENUS and insensitive mDII-ntd TOMATO into one reporter to rapidly quantify changes in auxin using fluorescence ratio. DR5v2 is composed of the *DR5* promoter and a novel binding site for ARF transcription factors designed to increase sensitivity and precision of auxin response visualization in *Arabidopsis* (Liao et al., 2015).

Rice exhibits divergent morphologies in root, shoot, inflorescence and flower tissue organization compared to dicotyledons. For instance, in *Arabidopsis*, a single primary root emerges from the embryo, later forming numerous lateral roots employing auxin-dependent initiation, patterning and emergence mechanisms (Lavenus et al., 2015). In contrast, rice develops a fibrous root system, composed of >100 crown roots bearing several lateral root types (Coudert et al., 2010). Auxin also regulates crown root (Inukai et al., 2005; Liu et al., 2005) formation and emergence in rice and also impacts lateral root formation (Liu et al., 2009). Similarly, in *Arabidopsis* floral meristems (FMs) initiate directly at the flank of IMs, and their formation is dependent on local auxin accumulation at the periphery of IMs (Yamaguchi et al., 2013). In contrast, rice exhibits a specialized inflorescence shape with primary and secondary branches, and spikelets attached on the branches (Zhang et al., 2013; Zhang and Yuan, 2014). To clarify the role of auxin during rice development, the *DR5*-GUS reporter was transformed into rice (Scarpella, 2003), to infer auxin distribution by analyzing GUS (β-glucuronidase) activities. However, the GUS reporter has low spatio-temporal resolution because of the longer protein turnover time of GUS protein and experimental variation in temperature, incubation time and pH, which frequently causes imprecise results of auxin location (Rahman et al., 2014).

In this study, we established and validated two auxin response reporter systems in rice: *DR5*-VENUS and *DII-VENUS*. Using these two reporters, we followed dynamic changes of auxin during rice development. This work describes new molecular tools for future auxin research in rice, but also provides the first insight in comparative auxin distribution and role in plant between monocot and dicot models.

## Materials and methods

### Plasmid construction and transformation

The *DII-VENUS* fragment containing the coding sequence for the degradation motif of the domain II of *Arabidopsis* AUX/IAA28 (AtIAA28) protein subcloned from *35s:: DII-VENUS* plasmid (Brunoud et al., 2012) was inserted into the binary vector pUBI::CAMBIA1301 (CAMBIA) using *Kpn* I and *Bam* HI restriction sites, under the control of maize ubiquitin-1 promoter. The *DR5*_*rev*_:*:*VENUS construct in pMLBART was composed of a generic synthetic promoter with nine repeats of core sequence (TGTCTC) reversely linked with CaMV minimal 35S promoter (Ulmasov et al., 1997; Friml et al., 2003), the triple VENUS sequence and the nuclear localization signal N7 (Cutler et al., 2000), which was harvested from Heisler et al. (2005). The two resultant vectors were transformed separately into rice japonica cultivar 9522 calluses with *Agrobacterium tumefaciens* EHA105 using *Agrobacterium*-mediated method (Hiei and Komari, 2008). We got 30 positive independent T0 transformants containing *DR5-VENUS*, Among these lines, 4 lines were identified as the homozygous plants showing similar and stable expression patterns during propagation. Among the nine positive T0 DII-VENUS lines, one line having the strongest and stable signals was selected for further analyses.

### Multiple sequence alignment and prediction of putative ARF binding sites

Amino acid sequences of 31 OsAUX/IAA members and AtAUX/IAA28 protein from Rice Genome Annotation Project (http://rice.plantbiology.msu.edu/) and TAIR (http://www.arabidopsis.org/), respectively, were aligned using MUSCLE 3.6, and then adjusted manually in GeneDoc 2.6. ARF binding sites among the 3000-bp promoter region of each *OsGH3* family was analyzed using PLANTPAN 2.0 (http://PlantPAN2.itps.ncku.edu.tw) (Chow et al., 2016).

### Plant growth and vibratome sectioning

Rice seedlings were grown vertically in sterile square petri dishes (Corning, 431301; 20 cm × 20 cm) under controlled conditions (day/night temperature of 28/25°C, a 12 h photoperiod, and a light intensity of 500 μEm-2s-1) for 3 days. Tissue parts of rice root, stem base, leaves and shoot apices were dissected and embedded in 3% agarose blocks (Lartaud et al., 2014). After solidification and reshaping, materials were cut into 70 μm slices in thickness with Thermo Vibratome 750. Agar parts of slices were carefully removed in water, and samples were quickly transferred on slides and immersed in a drop of 10% glycerol for imaging.

### Chemical treatments

For live imaging, 3-days old *DR5*-VENUS seedlings were treated for 1 day in 100 nM auxin transport inhibitor N-1-Naphthylphthalamic acid (NPA), and 3 days separately in 500 nM 1-Naphthaleneacetic acid (NAA) and 500 nM trans-zeatin (TZ) water solutions. For mRNA analysis, 6-days old wild-type seedlings were treated for 1.5 h in 1 μMNPA, 5 μMNAA, and 5 μM TZ water solutions, respectively.

### Root gravitropism assay

Firstly, rice seeds were sterilized using 50% bleach for 10 min with gentle shaking, and then washed for 6~7 times with sterile double distilled water. Seeds were dried for 3 min, then laid on half Murashige and Skoog (MS) solid medium and grown them vertically for 5 days. Following plate rotation to 90 degrees, time-serial pictures were taken at 30 min intervals automatically. Root tip angles were measured in ImageJ software.

### Sample preparation and microscope observation

Fluorescence images were taken on Zeiss LSM510 SP5 confocal, or TLSM 7MP/OPO two photon microscopy. For tissue organization observation, root tips were stained using 10 μg/ml Propidium Iodide (PI) solution for 10 min in dark and rinsed in double distilled water for 3 times, then included in low melting 0.5% agarose, mounted between a slide and a cover slip of 170 ± 1 μm for TLSM observation. Cell organization of rice vegetative tissues was visualized using chlorophyll autofluorescence. Fresh sections or intact tissues were immersed in a drop of 10% glycerol for LSM510 live imaging.

Under the SP5 microscope, Z-stacks were scanned every 1.5 μm in thickness and maximum projections were generated. For the TLSM, VENUS and PI emissions were collected in separate channels with excitation at 950 nm (Chameleon Ultra II) and 1,096 nm (Chameleon Compact OPO) with a gain set at 600 nm using 2PMT NDD and 2 PMT BiG detectors.

### Gene expression analysis

Root samples of 6-days old plants after drug or water treatment were collected instantly. After fixation in liquid nitrogen, samples were ground and then transferred into tubes filled with Trizol (Sigma). Total RNA was extracted using the traditional chloroform method, DNA was removed with DNA eraser reagent at 42°C for 2 min and cDNA was reverse transcribed from 1 μg total RNA by using Takara PrimeScript™ RT reagent Kit. Real-time qRT-PCR was performed on Bio-Rad CFX96 machine by the three-step method. Expression levels of those genes were normalized using those of *tublin* β*-4* and *ubiquitin 2* as the reference. Specific primers were in Supplementary Table S1.

### Immunostaining

Flower materials were fixed, wax-embedded and sectioned following the whole mount protocol (Paciorek et al., 2006). After clearing sections using Histoclear solution with increasing proportions of ethanol (100% Histoclear, 2:1 solution of Histoclear and absolute ethanol, 1:2 solution of Histoclear: 2ethanol, 100% ethanol), samples were rehydrated gradually, with ethanol 95, 70, 50, 30, and TBS buffer (100 mM Tris-HCl, 150 mM Nacl, pH: 7.5), 3~5 min for each step. The crosslink formed by paraformaldehyde was destroyed by treating slides for 30 min with target retrieval solution (DakoCytomation) at 33°C. After BSA solution (0.5% BSA, 0.02% Tween-20 in TBS) blocking slides for 1 h at room temperature, PIN1 proteins were detected by applying primary mouse monoantibody (1:1,000) obtained from Professor Klaus Plame (Pasternak et al., 2015) at 4°C overnight, and Alexa Fluor 488-conjugated goat anti-mouse secondary antibody (1:800) at RT for 2 h. Specific fluorescent signals were then captured through Leiss LSM510 confocal system.

## Results

### Rice genome has conserved auxin-responsive elements and auxin-interacting domain sequences

To reveal whether the auxin responsive element AuxRE or ARF transcription binding sites located at promoter regions of primary auxin responsive gene families in *Arabidopsis*, such as *GH3, AUX/IAA*, and *SAUR* (Abel and Theologis, 1996; Ulmasov et al., 1999; Chen et al., 2014) genes are conserved in rice, we searched for multiple AuxRE sites by scanning the 3,000-bp promoter regions upstream of translation start sites of 11 *OsGH3* genes. We observed that auxin responsive sequences (ARS, TGTCTC) were highly enriched in rice promoter regions of *OsGH3.3, OsGH3.5, OsGH3.12* (Supplementary Table S2), while no ARS was present within the *OsGH3.10* promoter, which are well in line with the responses of increased expression of *OsGH3.3, OsGH3.5, OsGH3.12*, and no detectable change in transcriptional level of *OsGH3.10* induced by auxin treatment (Jain et al., 2006b; Terol et al., 2006). Therefore, we decided to directly use the synthetic *DR5*_*rev*_ promoter containing ARS sequences to monitor auxin responsive expression in rice tissues.

The DNA fragment encoding the DII degradation domain of AtIAA28 was used in *Arabidopsis* auxin sensor *DII-VENUS* owing to its relatively long half-life (Brunoud et al., 2012). In rice, there are 31 AUX/IAA proteins (Jain et al., 2006a), and through the alignment of *Arabidopsis* IAA28 protein, we observed that rice AUX/IAA members share the consensus degron sequence GWPPV, and the conserved dipeptide KR between the first two domains (Supplementary Figure S1). Because little is known about the stability of rice AUX/IAAs *in vivo*, we generated the *UBI::DII-VENUS* via inserting the cDNA sequence between KQ (KR for other AtIAAs) and DII of AtIAA28 together with VENUS and nuclear localization signal N7 under the control of the maize ubiquitin-1 promoter (Supplementary Figure S2), which has been proved to have a relative stronger transcriptional ability in reproductive tissues than *CaMV35S* promoter (McElroy and Brettell, 1994).

### *DR5*-VENUS is applicable for the detection of auxin relocation and cellular level in rice

#### DR5-VENUS is sensitive to exogenous NAA treatment

**Figure 1:**
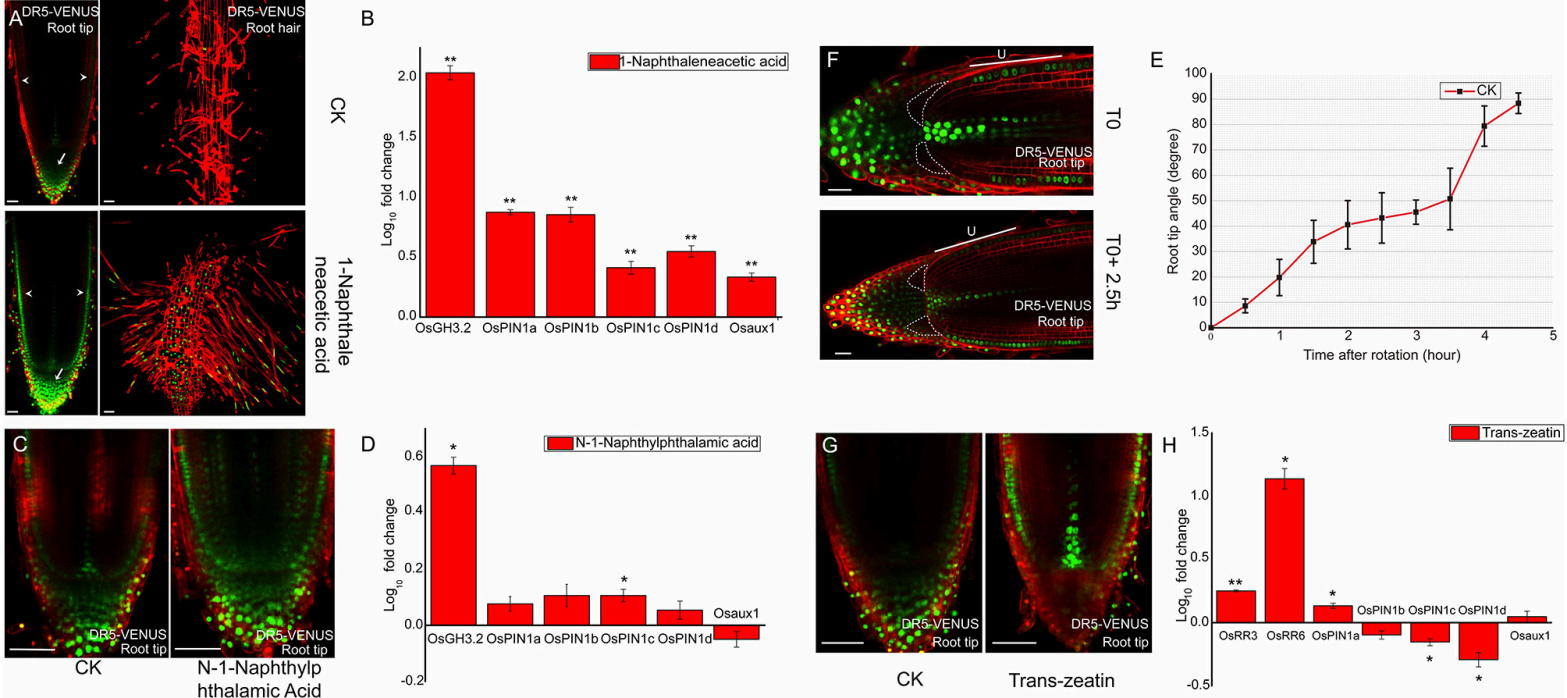
**Auxin responses to outside stimuli at ***DR5***-VENUS root tip. (A)** 500 nM NAA treated 3-days old *DR5*-VENUS rice seedlings for 3 days; images were taken at root tip and root hair zone. NAA raises auxin response at the epidermal (arrowheads), and columella cells (arrows). Scale bar: 50 μm. **(B)** Quantification of relative mRNA levels of auxin transporter genes after NAA treatment. Auxin responsive gene OsGH3.2 is used as positive control. All the data were expressed as mean ± SEM from three biological and three technical repeats. Statistically significant differences (Student's *t*-test, *P* < 0.01) between mock control (CK) and NAA treatment are indicated by two asterisks. Expression levels of all auxin transporters were increased after NAA treatment, including OsAUX1. **(C)** 100 nM NPA inhibition effect on *DR5*-VENUS rice primary root for 1 day. Auxin profile becomes much broader after NPA treatment and expands into cortex layers inside meristem. Scale bar: 50 μm. **(D)** Quantification of relative mRNA levels of auxin transporter genes after NPA treatment. OsGH3.2 is used as reference gene induced by auxin. All the data were expressed as mean ± SEM from three biological and three technical repeats. Statistically significant differences (Student's *t*-test, *P* < 0.05) are indicated by one asterisk. NPA has no effect on OsPIN1 genes except for OsPIN1c slightly induced by NPA. **(E)** Root gravitropism assay of rice wild type 9522 cultivar (CK). Root tip angle reaches up to 45 degrees at about 2.5 h after rotation in Rice. Root tip angles were calculated using image J software. All the data were expressed as mean ±SEM (Root Number = 8). **(F)** *DR5* signal redistribution at epidermis cell layer and root cap region under a gravity stimulus of 2.5 h. “U” represents the epidermis layer at upper side. *DR5*-VENUS signals at epidermis upper side are weaker under gravistimulation. Scale bar: 25 μm. White dotted lines highlight the lower region of lateral root cap where auxin accumulates. **(G)** *DR5*-VENUS signals at the root tip after 500 nM Trans-zeatin (cytokinin) application on 3-days old rice seedlings. Scale bar: 50 μm. Trans-zeatin induces relocation of auxin response from root cap to epidermis and cell initials inside meristem. **(H)** Quantification of relative mRNA levels of auxin transporter genes after cytokinin treatment. Type-A cytokinin responsive genes OsRR3 and OsRR6 are selected as positive control genes. All the data were expressed as mean ± SEM from three biological and three technical repeats. Statistically significant differences (Student's *t*-test, ^**^P < 0.01; ^*^P < 0.05) are marked out. OsPIN1c and OsPIN1d are significantly reduced while OsPIN1a slightly increased under CK treatment. **(A,C,F,G)** Red channel, propidium iodide; Green channel, VENUS.

To test the sensitivity of rice *DR5*-VENUS line and the authenticity of these auxin response, we treated the transgenic plants using active synthetic auxin NAA. Consistent with previous observation (Rahman et al., 2007), NAA inhibits rice primary root elongation in a dose-dependent manner (Supplementary Figure S3A). At the rice root tip under NAA treatment, the auxin reporter signal is visible in the root cap zone, outermost epidermal layer, as well as the root hair zone (Figure 1A, bottom panel), compared to the untreated control (Figure 1A, top panel). Moreover, with higher NAA concentrations, the signal at the root tip was gradually increased (Supplementary Figures S3B,C) confirming the dose-dependent response of *DR5*-VENUS to auxin levels. Consistent with the increased *DR5*-VENUS signal, exogenous auxin treatment enhanced the transcription of the auxin inducible gene *OsGH3.2* and auxin transporter genes *OsPIN1a, 1b, 1c, 1d*, and *OsAUX1* (Figure 1B). These results suggest that signal distribution of *DR5*-VENUS measures auxin presence *in situ* in rice. As application of exogenous auxin also induced quantitative changes in *DR5*-VENUS signal, these results suggest the reporter provides a reliable means to measure auxin levels.

#### The auxin transport inhibitor NPA disrupts DR5-VENUS pattern

To further assess authenticity of the auxin response in the *DR5*-VENUS marker line, we blocked polar auxin transport of the marker line using the auxin transport inhibitor NPA. This treatment caused the auxin gradient between epidermis and inner tissues to disappear, the intensity of the auxin maxima located in root cap and vasculature to weaken, and the VENUS fluorescent signals in the QC also became broadly diffuse (Figure 1C and Supplementary Figure S4). As to the auxin transporters, the expression level of *OsPIN1c* gene was statistically up-regulated, most probably due to local auxin accumulation in response to NPA treatment (Figure 1D). Our results suggest that the *DR5*-VENUS reporter can be altered indirectly by disrupting polar auxin transport.

#### Dynamic changes in DR5-VENUS at the root tip following a gravity stimulus

Additionally, we used *DR5*-VENUS to monitor dynamic changes of auxin gradients during root gravitropism. After placing the rice root horizontally, the root tip took approximately 4.5 h to return to its vertical position (Figure 1E). After 2.5 h, the most notable asymmetric pattern of *DR5*-VENUS activation was first seen when the root angle reached 45°. Weak *DR5*-VENUS signals in the lower half of lateral root cap cells adjacent to columella cells (highlighted with dotted lines) appeared, which were dramatically increased compared with their upper counterparts. Besides, in the meristematic zone, the fluorescent intensity at the upward side of epidermis was largely weakened, while the signals underneath remained stable (Figure 1F and Supplementary Figure S5), which was complementary to that of *DII-VENUS* (Supplementary Figure S6), but differs from that of *Arabidopsis* in which *DR5*-VENUS expression was pronouncedly elevated in lower epidermal cells after gravistimulation (Band et al., 2012; Brunoud et al., 2012). These results suggest the existence of a complex pattern of auxin distribution within root cap and epidermal tissues in rice following gravistimulation. Moreover, lateral root cap and epidermis formation results from distinct initials in monocotyledons, compare to dicotyledons where the latter differentiates from a common ones (Clowes, 1994). The existence of a root cap junction clearly separating root cap and meristem in monocotyledons may be responsible for the divergent pattern of auxin relocation in rice roots compared to *Arabidopsis* (Rebouillat et al., 2008; Wang L. et al., 2014).

#### Cytokinin indirectly induces changes in DR5-VENUS spatial expression

In agreement with observations in *Arabidopsis* (Ruzicka et al., 2009; Shimizu-Sato et al., 2009; Shen et al., 2014), cytokinin application caused a significant increase in *OsRR3, OsRR6*, and *OsPIN1a* transcript abundance and an up regulation of auxin response in the epidermal, stele and quiescent center (QC) cells. This treatment also decreased auxin content at the root cap zone through down-regulating expression of two auxin carriers *OsPIN1c* and *OsPIN1d* (Figures 1G,H), confirming the existence of crosstalk between auxin and cytokinin in rice roots. This result is consistent with the antagonistic effect of gene expression profiles related to these two phytohormones in the root apex (Takehisa et al., 2012).

### Auxin distribution during rice leaf development

**Figure 2:**
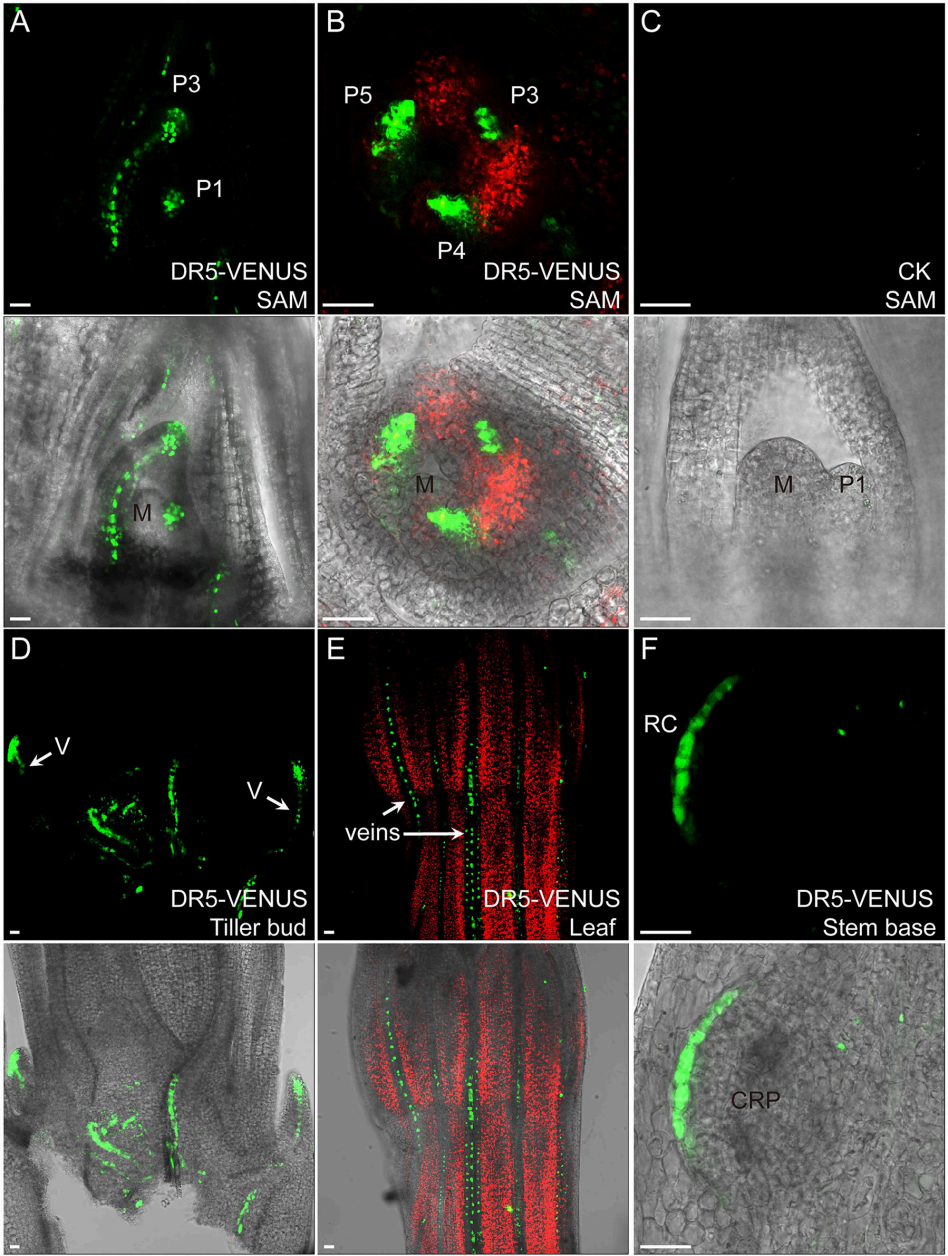
**Auxin expression sites in rice vegetative tissues**. In **(A,B)**, auxin highly accumulates in leaf primordia but is lacking in meristem center. Medial longitudinal view of SAM in **(A)** and top view of SAM in **(B)**. The median longitudinal view of 9,522 SAM is used as a control **(C)** “P” marks leaf primodium, and numerals denote first, third, fourth, fifth leaf primordia, respectively. “M” indicates meristem center. **(D)** *DR5*-VENUS expression at tiller buds and their connected vasculature (V, arrows). **(E)** *DR5*-VENUS expression in veins (arrows) of mature leaf surface. **(F)** *DR5*-VENUS expression at root cap and root cap initials (RC) of a crown root primodium (CRP) at rice stem base. Scale bar: 25 μm. **(A–F)** are images under the fluorescent field. Red channel, chloroplast autofluorescence; Green channel, VENUS.

**Figure 3:**
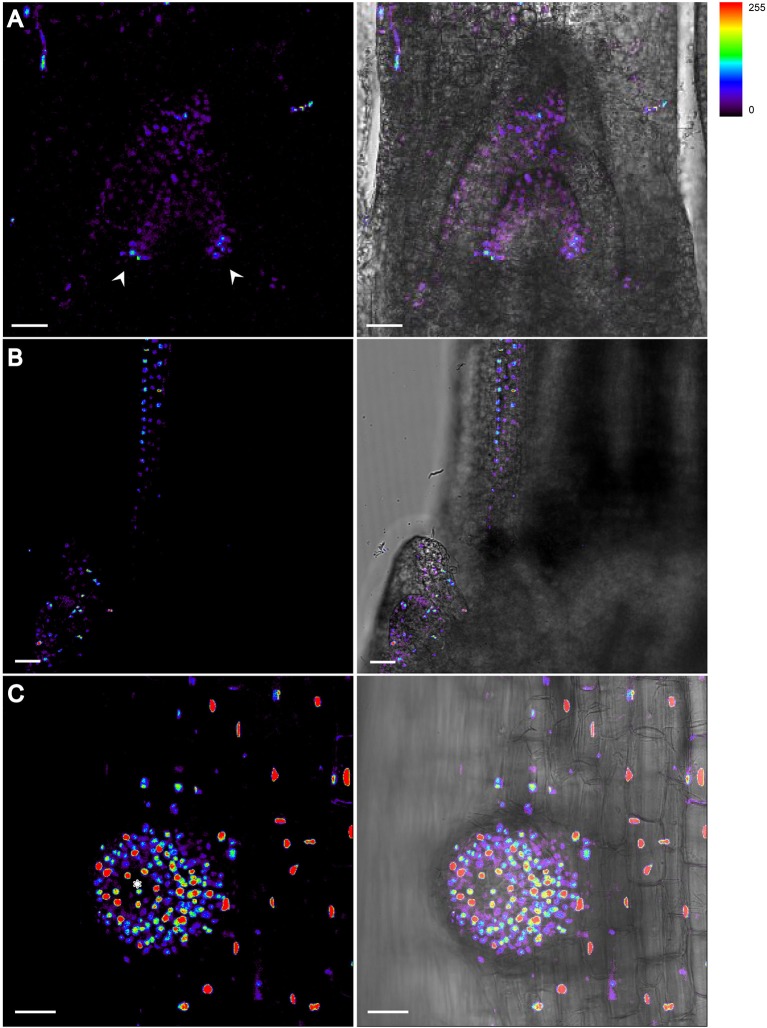
*****DII-VENUS*** profiles in vegetative organs of Rice. (A)** Auxin level is lower at apical meristem and axils. Arrowheads indicate the strongest DII-VENUS signals at both axils and thus the lowest auxin level. **(B)** Auxin reaches the maximum at the tip of tillering bud where DII signals are undetectable. **(C)** Highest auxin expression level is detected at the tip of root cap region of an emerging lateral root. Asterisk indicates the root cap region with no detectable DII signal. The level of *DII-VENUS* is shown in pseudocolor from blue to red (Spectrum LUT bar in the top right). Blue, no signal; Red, strong saturated signal intensity. Scale bar: 25 μm.

In the shoot apical meristem (SAM), *DR5*-VENUS signals were only detected at the adjacent leaf primordia, while *DII-VENUS* was found at the apical meristem (Figures 2A–C, 3A), especially enriched at both leaf axils, suggesting that the rice SAM represents a zone of an auxin limitation, at least at a certain period of vegetative development, instead of being an auxin sink, contrasting that reported in *Arabidopsis* and suggesting an intricate auxin mechanism in regulating rice SAM function. Given the presence of *DR5* expression in adjacent leaf primordia, auxin may be locally synthesized and contribute to leaf growth (Qin, 2005; Cheng et al., 2007; Li et al., 2008). Consistently, auxin depletion at leaf axils of *Arabidopsis* and tomato has been shown to be essential for axillary meristem formation (Wang Q. et al., 2014).

At the rice stem base, we observed that the *DR5*-VENUS signal accumulated in vascular tissues and apices of nearby tillering buds, leading us to speculate that auxin is transported from newly formed leaves basipetally down through the vasculature system for suppressing their outgrowth (Figures 2D, 3B). Consistently external IAA application inhibited the growth of tiller buds by decreasing the endogenous level of cytokinin in rice (Liu et al., 2011).

Our reporter analysis also suggested that auxin may be also involved in leaf vein development and root cap formation of emerging crown root at the stem base (Figures 2E,F). Supportively, previous research using rice mutants showed that the impaired polar auxin transport induced defects in leaf vascular patterning (Qi et al., 2008). Moreover, auxin can affect crown root formation in rice by regulating the expression of *CRL1* gene through transcription factor ARF (Inukai et al., 2005).

### Auxin plays a key role in lateral root development and emergence

**Figure 4:**
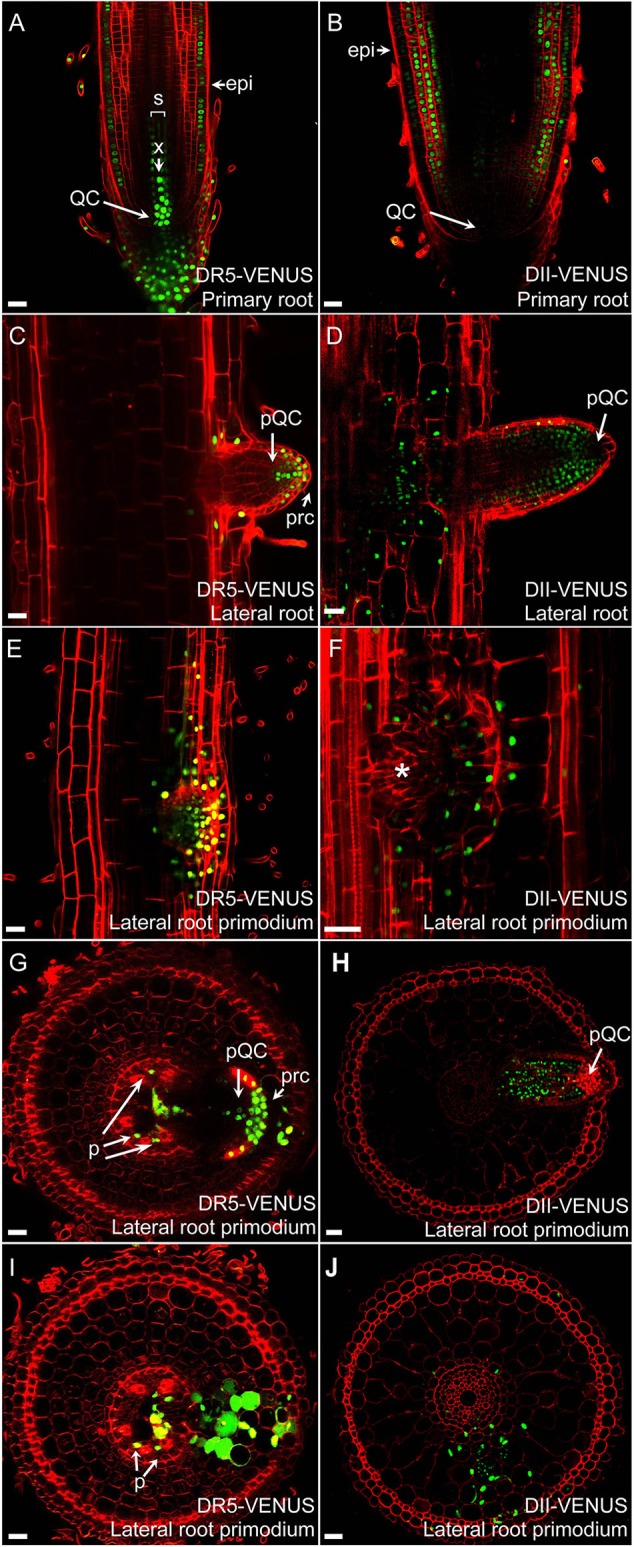
**Auxin distribution in rice roots**. Auxin distributions in radicles of *DR5*-VENUS **(A)** and *DII-VENUS* **(B)** transgenic lines. Expression profiles of *DR5*-VENUS and *DII-VENUS* are fully complementary. Auxin maxima is visible in QC, columella cells, initials cells, X, and Epi while virtually absent from cortex, ground tissues and lateral root cap. In developing lateral roots **(C,D)**, auxin accumulates inside QC, root cap and in flanking zone of lateral root. During early LRP formation **(E,F)**, auxin is visible inside developing lateral root meristem. Transverse sections show auxin localization at median planes **(G,I)** and nearby layers **(H,J)** of lateral root primodium ready to emerge from primary root. Auxin is visible in phloem (p) and again in pQC, prc and lacking inside lateral root apical meristem. Epi, epidermis; X, central metaxylem; QC, quiescent center; pQC, putative quiescent center; prc, putative root cap; p, phloem. Asterisk indicates no signal inside lateral root primodium. Red channel, propidium iodide; Green channel, VENUS. Scale bar: 25 μm.

Rice develops a much larger and ramified root architecture compared to *Arabidopsis* (Chu et al., 2013; Wang L. et al., 2014; Kochian, 2016). *DII-VENUS* and *DR5*-VENUS reporters revealed that auxin forms maxima at the root cap, putative QC, stem cells and vasculature. Moreover, we found a relative higher signal in the epidermal layer of the meristematic and elongation zone in the primary roots, compared with *Arabidopsis* (Figures 4A,B), which was also confirmed by the auxin response at root hair and root surface at the differentiation zone (Figure 1A and Supplementary Figure S3C). Strong *DR5*-VENUS signals in central metaxylem, protoxylem and companion cells of the phloem were also clearly visible (Supplementary Figure S7).

**Figure 5:**
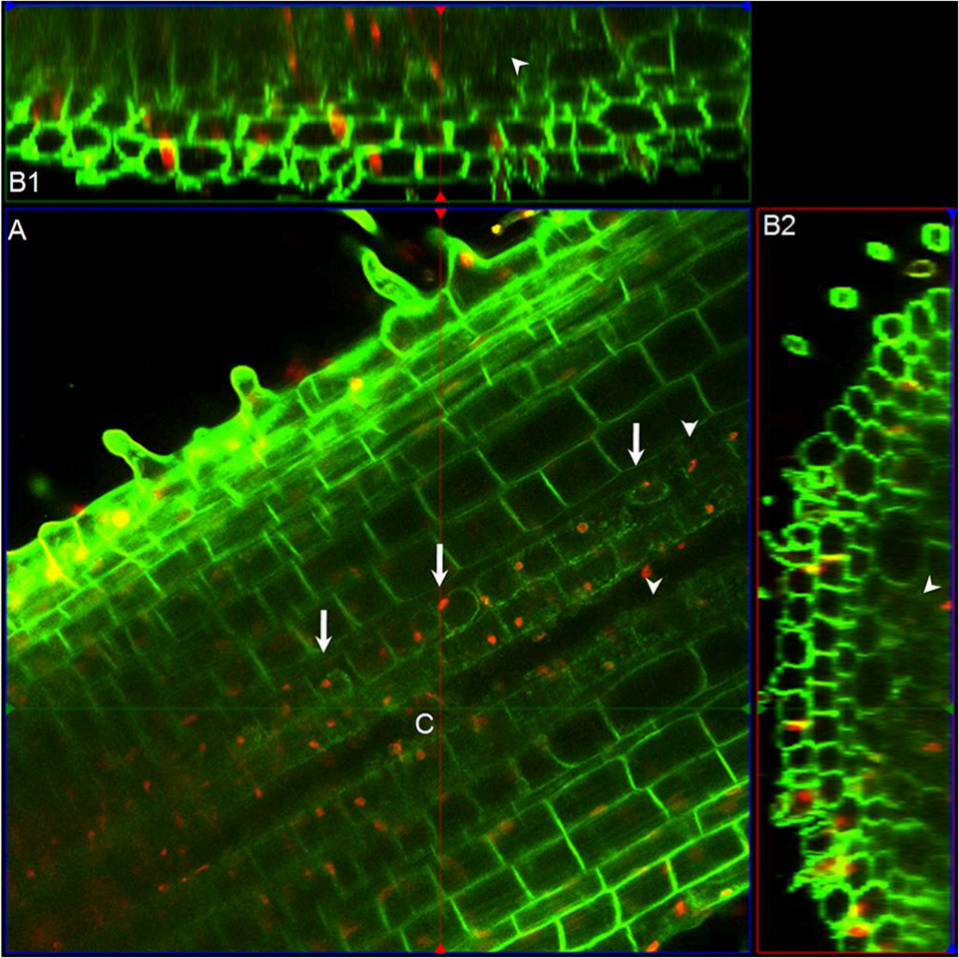
**Morphological features of cortex cells in differentiation zone of rice roots. (A)** Longitudinal section above the median plane inside the primary root of Lti6a:CFP;H2B:mCherry transgenic seedlings. Chromatin in cell nucleus was marked in red, and cell membrane was marked in green. **(B1)** and **(B2)** are the sagittal and radial views at the intersection point **(C)** obtained with the ortho view function of Zeiss Zen software. Arrows indicate the membrane retractation of cortex cells, and arrowheads points out the cortex cells with unrecognizable cell outlines. Images were taken and analyzed using ZEN (Zeiss) and Fiji software. Red channel, mCherry; Green channel, CFP.

Strong *DR5*-VENUS signals were also observed in rice lateral roots at the root tip (Figures 4C,D, 3C), in LRP (Figures 4G–J) as well as in cortex cells overlying LRP (Figures 4E,F). Surprisingly, in these cells, the *DR5*-VENUS signal was cytoplasmic instead of the always-observed nuclear localization of the VENUS signal. We then used Lti6a:CFP;H2B:mCherry transgenic lines to follow cortex cell differentiation in mature root parts (C. Périn and M. Ingouff unpublished) (Zhang et al., 2011; Howe et al., 2012). In these lines, plasma membrane and chromatin are marked by CFP and mCherry, respectively. Cortex cells in the differentiation zone of rice roots were undergoing programmed cell death, with indistinct cell borders (arrowheads), membrane retraction (arrows) and abnormal disaggregating nuclei (Figure 5), paving the way for the LRP to later emerge. This result suggests there is an increase in auxin level in cortex cells surrounding LRP that may be responsible for the collapse of cortex cells during root organ emergence in rice, to be compared with the cell wall breakdown triggered in endodermal cells during LRP emergence in *Arabidopsis* (Peret et al., 2009).

### Auxin distribution is associated with rice inflorescence branching

**Figure 6:**
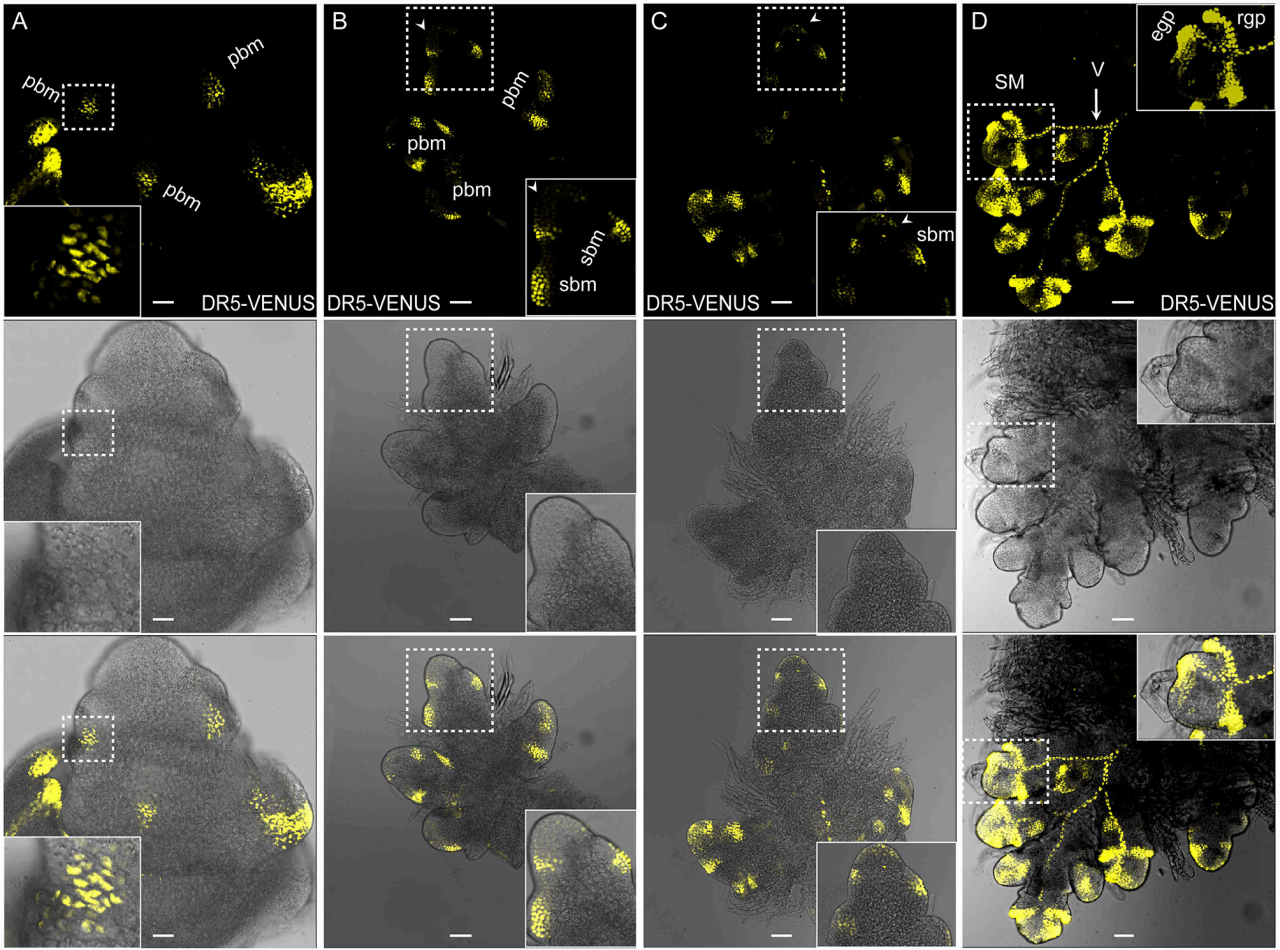
**Auxin distribution during rice inflorescence branch formation using ***DR5***:VENUS. (A)** Primary branch meristems (pBMs) are marked out at the rachis. Scale bar: 25 μm. **(B)** Late pBM, suggesting emerging sites for secondary branch meristem (sBM). Relative weak signals at the first layer were also observed (arrowheads). Inserted image is the magnified version of the pbm in the process of generating two incipient sbms in the dotted region. Scale bar: 25 μm. **(C)** Developing sBMs. Signals at the first layer were observed (arrowheads). Inserted image is the magnified region in the dotted box. Scale bar: 50 μm. **(D)** Fluorescence in spikelet meristem (SM) shows strong auxin response at glume primordia (including rgp and egp) and the presumptive vascular strands (V, arrow). rgp, rudimentary glume primodium; egp, empty glume primodium. Scale bar: 50 μm. Yellow channel, VENUS.

**Figure 7:**
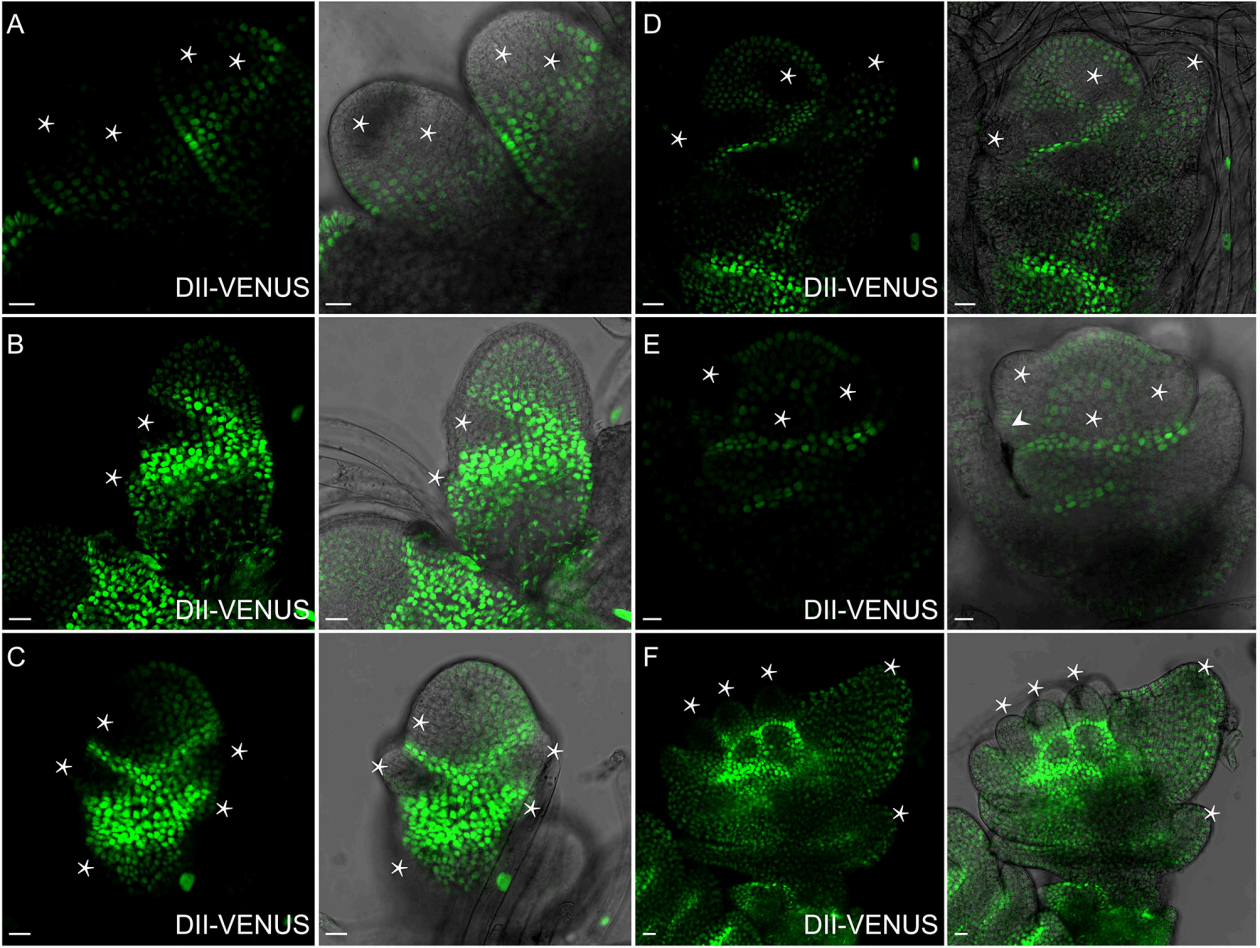
**Auxin distribution during flower formation using ***DII-VENUS*** sensor. (A)** Secondary branch primordia formation at the primary branch. Auxin reaches maxima at these potential sites (Asterisks). **(B)** Strong auxin levels observed in glume primordia (Asterisks), including rudimentary and empty glume primordia. **(C)** Lemma primordium marked out with strong auxin (Asterisk). **(D)** High auxin levels at palea, lemma and floral meristem (Asterisks). **(E)** Three stamen primordia indicated by asterisks. Lodicule primordium (arrowhead) besides the left stamen primordium has lower auxin level. **(F)** Relatively strong auxin signals are visible in young stamens and apices of glume primordia (Asterisks). Green channel, VENUS. Scale bar: 15 μm.

**Figure 8:**
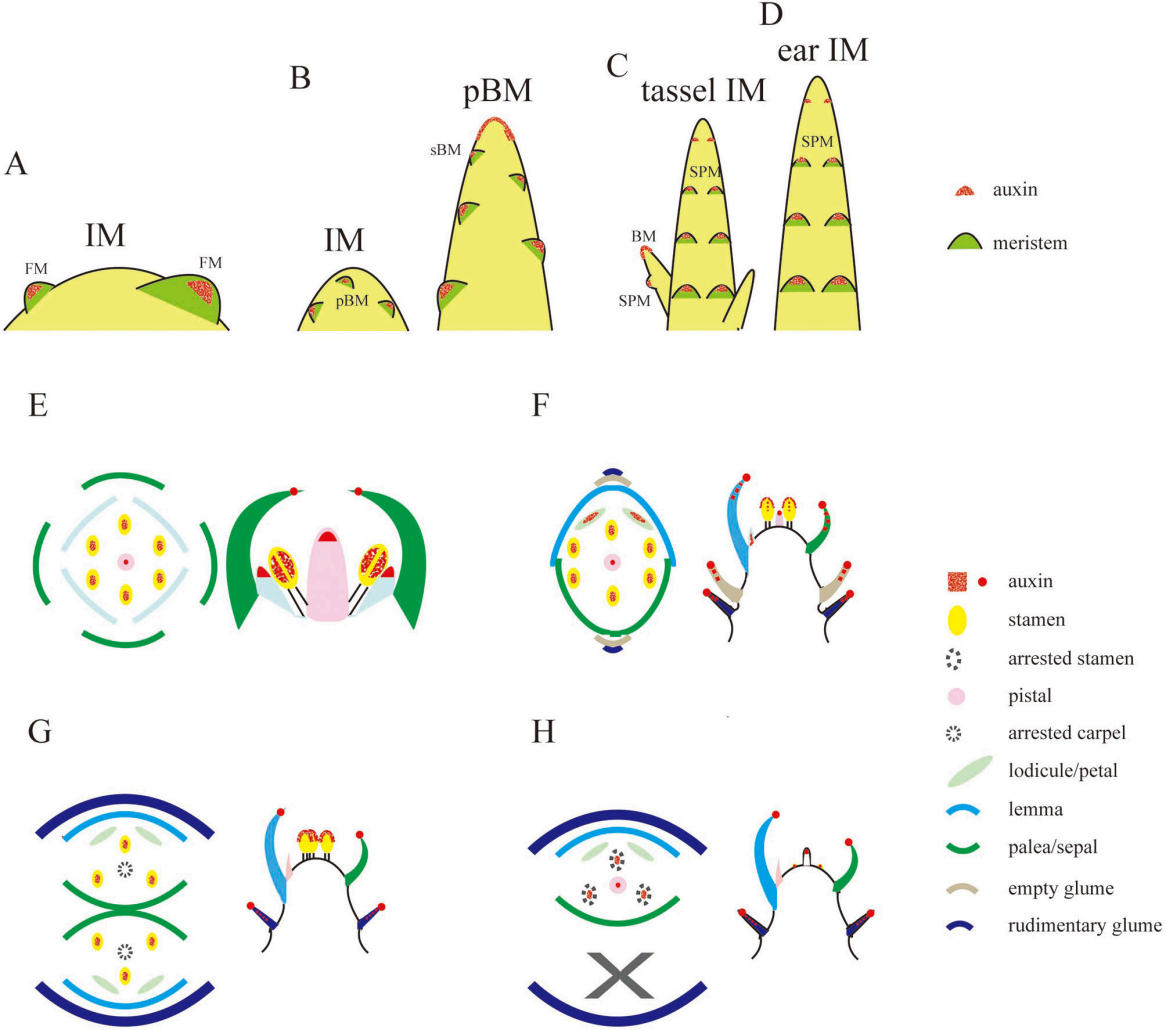
**Schematic representations of auxin levels during reproductive development. (A–D)** Auxin observation during the transition of IM in *Arabidopsis* **(A)**, rice **(B)**, and maize **(C,D)**. Auxin is only seen at the first layer of BM, including pBM and sBM, floral meristem (FM), and spikelet pair meristem (SPM). **(E–H)** Illustration of auxin distribution at floral meristem **(E)** in *Arabidopsis* or SM **(F)** in rice or male **(G)** and pistillate **(H)** SMs in maize. Sites in red only represent the potential auxin locations during the floral meristem development, and do not mean all of them can be observed at the same developmental stage. Rice tissues not being marked in red **(F)** means there is not auxin signals observed, and those in **(E,G,H)** only denote sites that have not been reported in literature (Aloni et al., 2006; Gallavotti et al., 2008; Yang et al., 2013; Eveland et al., 2014; Gallia et al., 2015).

Local auxin accumulation is required for reproductive organ initiation in *Arabidopsis* (Reinhardt et al., 2000; Benkova et al., 2003; Heisler et al., 2005; Yamaguchi et al., 2013), however the role of auxin distribution during rice flower development remains unclear. Specifically during the rice inflorescence formation, highly branched architecture is mainly produced from the inflorescence meristem (IM) (Zhang et al., 2013). In Figure 6, three new potential sites for the coming primary BMs had the obvious *DR5*-VENUS signals at IM (Figure 6A). While, as the elongation of the primary BMs, auxin response was shifted to the first several layers of BMs, and the locations where several secondary BMs were going to be formed (Figures 6B,C, 7A). Consistently, auxin response were also observed in the first layer of the BMs of maize tassel IM (Gallavotti et al., 2008), although the inflorescence morphology of rice differs from that of maize (Figures 8B,C). Notably, no obvious accumulation of *DR5*-VENUS signal was documented in the first layer of *Arabidopsis* BMs, and maize ear IMs which produce floral or spikelet pair meristem directly without generating branching meristem (Gallavotti et al., 2008; Gallia et al., 2015; Figures 8A,D), suggesting that auxin maximum at the first layer of BMs represents a sign for inflorescence branching. After secondary lateral branches are generated, the SM at the terminus of primary branch and others at secondary branches are initiated in succession, where auxin was traced at the developing vasculature of inflorescence, and also in primitive and maturing glume primordia (Figure 6D). These results suggest that auxin accumulation is a key determinant of rice inflorescence morphogenesis, particularly the formation of the characteristic branches.

### Auxin distribution in rice spikelet

**Figure 9:**
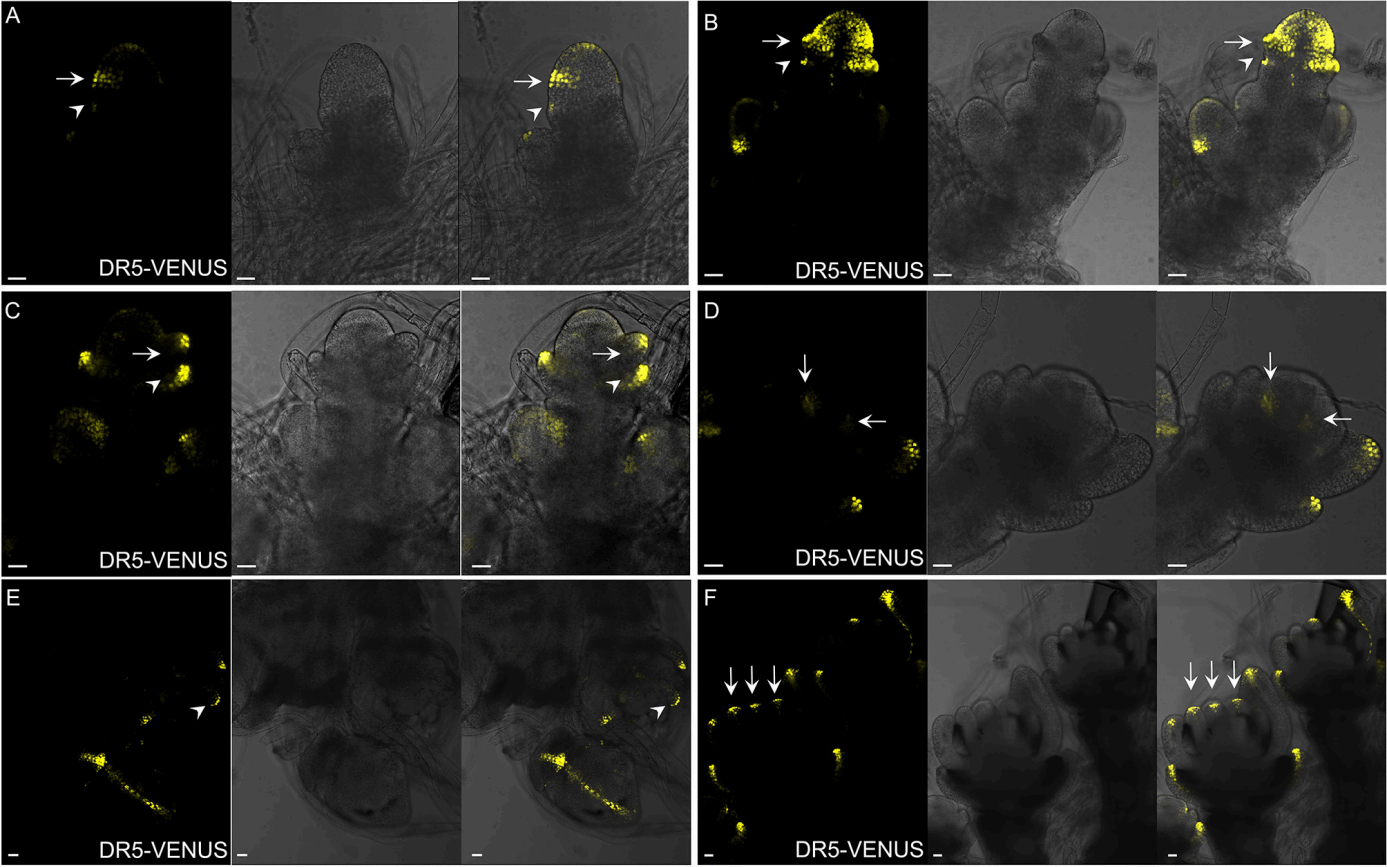
**Auxin distribution during spikelet development using ***DR5-***VENUS. (A)** Developing SM, during the formation of glume primordia. Signals are firstly observed at the first cell layer of SM and glume primordia, including rudimentary glume primodium (arrowheads) and empty primodium (arrows). **(B)** Strong auxin level in empty glume primordia (arrows and arrowheads) and SM. **(C)** At stage 3 of spikelet development, lemma primordium (arrows) is formed. Arrowhead indicates elongated empty glume. **(D)** Two lodicule primordia (arrows) with weak auxin response. **(E)** Strong auxin response observed at the first layer of stamen primodium (arrowheads), and vasculature of palea and lemma. **(F)** Longitudinal observation shows presence of strong auxin levels (arrows) inside stamen. Yellow channel, VENUS. Scale bar: 25 μm.

Spikelet is a unique and fundamental structure within grass inflorescences, which bears glume instead of petal structures enclosing the floret (Zhang and Wilson, 2009; Zhang and Yuan, 2014). Unlike *Arabidopsis* (Figure 8E), SMs of rice produce a pair of glume primordia at the very onset (Itoh, 2005), during which auxin is limitedly expressed at cells of the top joint zone where the rudimentary glume attaches to the meristem (arrowhead), the incipient site for the sterile lemma (arrow), and the first cell layer of the SM (Figure 9A). With the growth of a pair of sterile lemmas, auxin response was seen at the transition zone of the rudimentary glume (arrowhead), the floral meristem, and sterile lemma primordia (arrow) (Figures 9B, 7B), which are totally absent in maize flower.

**Figure 10:**
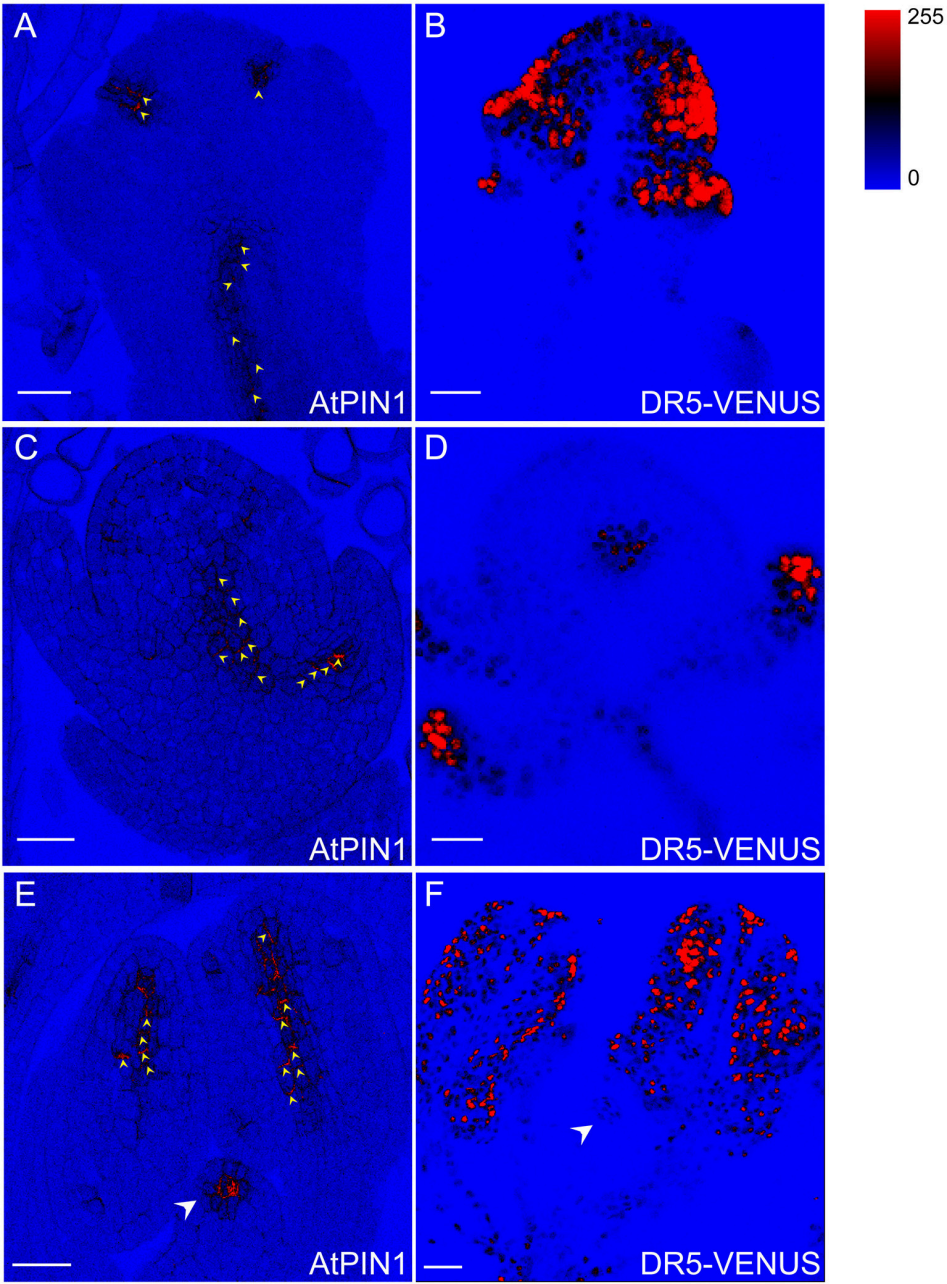
*****DR5***-VENUS overlaps with PIN1 protein localization in spikelets**. PIN1 immunolocalization (arrowheads in **A,C,E)** and *DR5*-VENUS signals **(B,D,F)**. PIN1 proteins **(A)** are detected at incipient sites of lemma and palea primordia **(B)**. At stage 4 of spikelet development, auxin transported by PIN1 **(C)** accumulates in glume primordia **(D)**. PIN1 location **(E)** in inner vascular bundles of stamen and pistil primodium (arrowheads) paves the way for auxin flow to pollen and pistil formation **(F)**. The level of *DR5*-VENUS/Auxin **(B,D,F)** and Alexa Fluor 488 **(A,C,E)** are shown in pseudocolor from blue to red (Spectrum LUT bar in the top right). Blue, no signal; Red, strong saturated signal intensity. Scale bar: 25 μm.

In contrast to those in maize spikelet pairs, rice florets are bisexual since initiation (Figures 8F–H). Among rice spikelet organs, the lemma is the first one appeared showing a strong *DR5*-VENUS signals at the apex, which was verified by complete exclusion of *DII-VENUS* expression at this region, and relatively low auxin at the first several layers of the meristem (Figures 9C, 7C), then the palea emerged out at the location where *DII-VENUS* signal was invisible (Figure 7D). Comprehensive analysis of lodicules showed that *DR5*-VENUS expression at lodicule primordia was detectable but relatively weak (Figures 9D, 7E). At the earlier stage, an auxin response was observed located at the first layer of the stamen primordia from the top view (Figures 9E, 7E), and at the inner vascular tissues of stamens seen from the longitudinal direction (Figures 7F, 9F), suggesting auxin may participate in rice anther development (Qu et al., 2014). In addition, *DR5*-VENUS signals were detectable at the stamen and pistil primordia (Figures 9E,F, 10E,F), which were also reported in maize unisexual floret (Gallavotti et al., 2008), although the development of gynoecia in maize tassel flowers, and stamen in ear flowers became a complete abortion, suggesting that auxin is essential for floral organ initiation instead of growth. Taken together, our observations suggest that auxin signaling may be essential for rice spikelet organ development.

### Auxin transport in rice spikelet organs

Auxin flow is achieved via specific transport proteins, including influx carriers AUX1/LAXs that determine in which tissues the hormone accumulates (Band et al., 2014), and polar efflux carriers PINs whose orientations can infer the intercellular direction of auxin movement (Wisniewska et al., 2006). To probe the relationship between rice flower development and auxin transport, sub-cellular localization of PIN1 homologs in rice (Supplementary Figure S8) were determined using immunostaining of AtPIN1 antibody applicable in rice (Pasternak et al., 2015; Figures 10A,C,E). After the formation of two empty glumes, the incipient sites of lemma and palea primordia were specified by PIN1 polar localization which may direct auxin movement through the basal vasculature (Figures 10A,B). Moreover, PIN1 localization overlapped with the spatial distributions of *DR5*-VENUS in spikelet primordia at stage 4 when the palea primodium formed, denoting that the auxin maxima at the spikelet primordia may be generated by the PIN1 action (Figures 10C,D). At the final stage of spikelet development, PIN1 exhibited strong expression in inner vascular bundles of anthers, suggesting a large amount of auxin possibly being delivered to young pollen grains (Feng et al., 2006). The PIN1 and auxin signals also remained in anther filaments during vascular tissue differentiation (Figures 10E,F). Besides, PIN1 signal was also visible at pistil primordium (Figure 10E), demonstrating that auxin may have function in affecting ovule development (Wu et al., 2015).

**Figure 11:**
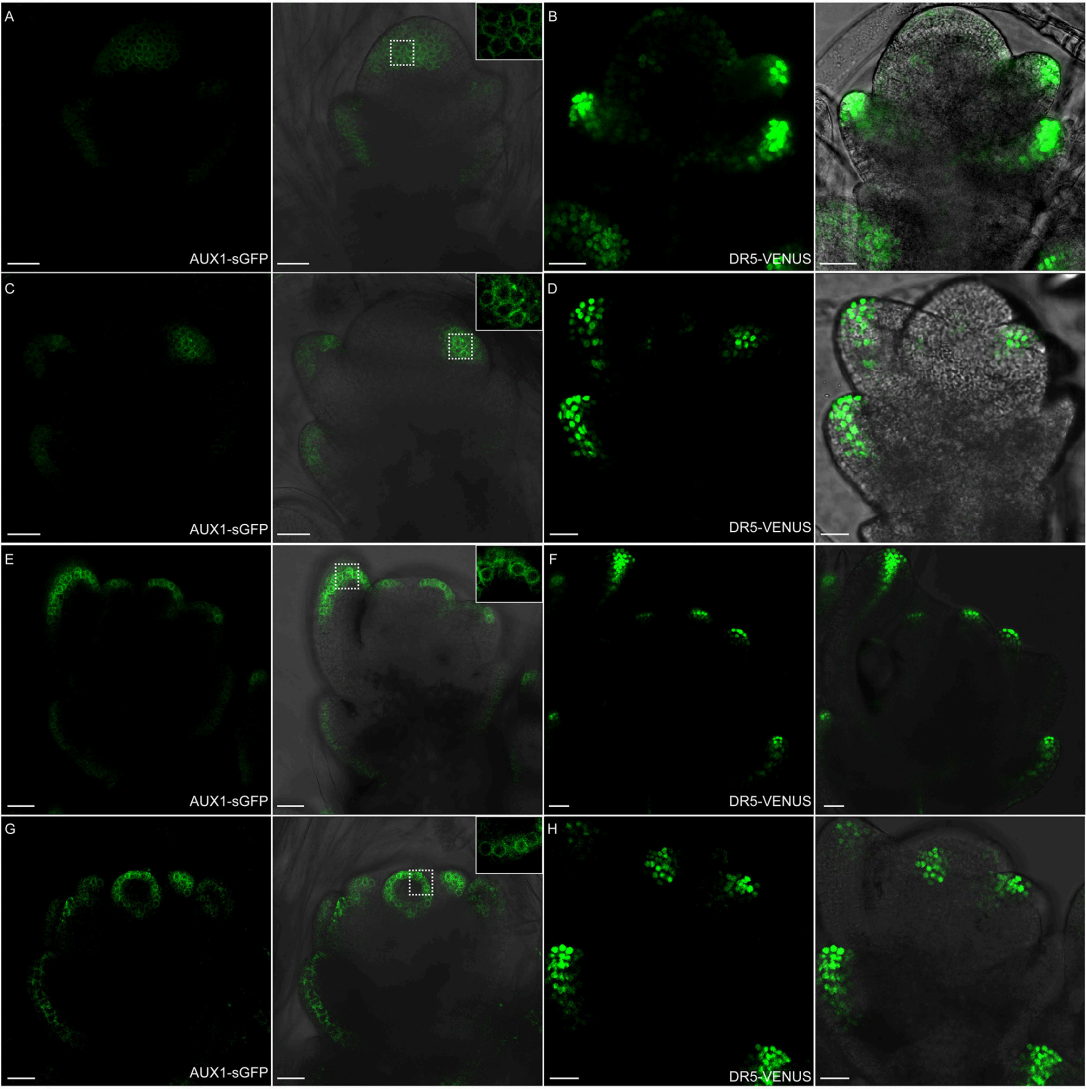
**OsAUX1 protein localization overlaps with auxin maxima during floral development. (A,B)** Spikelet meristems of ProOsAUX1:OsAUX1-sGFP transgenic lines are observed under confocal microscope. At stage 3, OsAUX1 is expressed in the floral meristem, including lemma and glume primordia, where *DR5*-VENUS signals are also present. Scale bar: 25 μm. **(C,D)** At stage 4 of spikelet development, both OsAUX1 and auxin are strongly expressed in palea primodium. Scale bar: 25 μm. **(E,F)** OsAUX1 is observed at lemma and first several layers of stamen primordia, overlapping with *DR5* expression sites. Scale bar: 25 μm. **(G,H)** OsAUX1 also has a relative high expression level at carpel primodium at stage 7. Scale bar: 25 μm. The closeup view in **(A,C,E,G)** are magnified pictures of signals visible inside dotted squares. Green channel in **(A,C,E)**, GFP; Green channel in **(B,D,F)**, VENUS.

Auxin uptake depends on OsAUX1 (LOC_Os01g63770) permease that modulates root initiation and elongation in rice (Yu et al., 2015). Using ProOsAUX1:OsAUX1-sGFP transgenic lines, we observed *OsAUX1* specific expression in rice floral tissues (Figure 11). Strong OsAUX1-sGFP accumulation was visible in floral primordium after the emergence of lemma primordium (Figures 11A,B), and the subsequent palea primordium (Figures 11C,D). The OsAUX1-sGFP signals were also seen in cells at the first several outer layers of stamen primordia (Figures 11E,F), and carpel primodium (Figures 11G,H), which overlapped with *DR5*-VENUS signals previously observed. Taken together, uniformly sub-localized OsAUX1-sGFP signals at cell membranes (see the magnified zones in Figure 11) coincided well with *DR5*-VENUS signals (Figures 10, 11), implying that PIN1 and OsAUX1 work together to convey and redistribute auxin during rice floral organ formation.

## Discussion

We demonstrated that two traditional auxin reporters *DR5*-VENUS and *DII-VENUS*, proven useful in *Arabidopsis* and Maize (Gallavotti et al., 2008), are also capable of revealing auxin distribution in rice. Similar hormone distribution maps at high spatial and temporal resolution were developed in all three experimental model plants, confirming the importance of polar auxin transport in regulating plant morphogenesis both in dicot and monocot species.

The response efficiency of synthetic *DR5*_*rev*_ promoter *in vivo* is higher in rice, which has broader expression profile compared to that in *Arabidopsis*. At rice primary root apex, besides marked signals at QC, columella cells and xylem, additional ones were also found at the epidermis layers of meristematic and elongation zones, and also within phloem cells at maturation region, which were invisible in *Arabidopsis DR5*-GFP or GUS lines (Figure 2A and Supplementary Figure S7), while partially of them were supplemented by using another auxin responsive element *DR5v2* (Liao et al., 2015). Through identifying AuxRE sites in 3000-bp promoter regions of auxin early responsive gene family GH3 in rice and *Arabidopsis*, strikingly, we found out that the occurrence of canonical sequence TGTCTC in rice is dramatically increased (Supplementary Figure S9). We hypothesize that TGTCTC containing sequences may have a greater contribution ability to the auxin response through increase of ARF binding level *in vivo* in rice. Thus, screening the natural promoter conditions of auxin responsive gene families is probably a much more advisable strategy before transforming it into other plant species.

For the first time, this study provides a clear evidence that auxin plays a crucial role in rice flower formation. The overlapping localization patterns suggest that, *DR5*-specified auxin and its transporters PIN1, OsAUX1 signals are capable of providing positional information for flower primodium initiation (Figures 9, 10). Two weeks of NPA (30 μM) treatment during rice transition phase from vegetative to reproductive growth brought out yellow sterile inflorescence without any spikelet, at the same time, longer time and higher level of NPA (50 μM) adoption was lethal to rice plants, with deformed inflorescence arrested inside (data not shown), while IAA or NAA treatment prompted shoot apex differentiation into flower initials, further advanced rice flowering (Sircar and Kundu, 1955). However, rice *Osaux1* T-DNA mutants present out inconspicuous defects in spikelet structure or fertility, which may be explained by the genetic redundancy of AUX1-like gene family in rice. Therefore, local auxin gradient formed by auxin polar transport is required for rice flower organogenesis.

In this work, combining with *DR5*-VENUS, we use *DII-VENUS* patterns as negative controls for well defining auxin distributions in rice. *DII-VENUS* labels out strong auxin signals with minimum of florescence, profiles of which are quite complementary with *DR5* signals in almost all conditions and most notably during rice spikelet development (Figures 5–7). However, in few conditions at specific tissues, *DII-VENUS* reporter doesn't work well, for example in root (Figure 4) and shoot apical meristem (Figure 3A), a feature possibly caused by limited expression abilities of maize ubiquitin-1 promoter in these tissues. We hypothesize that it will be better if we replace domain II fragment of IAA28 *Arabidopsis*, which might possess differentiated stability and half-life characteristics in rice, with rice-specific ones, although the key residues for auxin interaction are considered generic and could be transformed into any plant species (Dreher et al., 2006; Zhang et al., 2016).

In conclusion, comparison of auxin localization and dynamic relocation between *Arabidopsis* and rice could help shed light on the auxin functions in angiosperms; these two biosensors represent important tools to understand the auxin signaling pathway in diverse rice developmental processes by transformation or genetic crossing method, but also to further reveal the strong link between auxin flow and agronomical traits of interest like aerial and root architecture or yield.

## Author contributions

WL, ZY, DZ, and JY designed the experiments. JY performed the experiments. QM and GH assisted in immunostaining technique. CP, CB, AM, and MI generated and analyzed the Lti6a:CFP;H2B:mCherry data. JY, WL, CP, and MB analyzed the data. JY, WL, and DZ wrote the article.

### Conflict of interest statement

The authors declare that the research was conducted in the absence of any commercial or financial relationships that could be construed as a potential conflict of interest.
