# Supplementary material for: Dynamic Regulation of Auxin Response during Rice Development Revealed by Newly Established Hormone Biosensor Markers
Source: Front Plant Sci. 2017 Mar 7;8:256. doi: 10.3389/fpls.2017.00256 (PMC5339295; doi:10.3389/fpls.2017.00256)
Supplement: Supplementary file 1 [file DataSheet1.doc]

**Supplementary Figures**

**
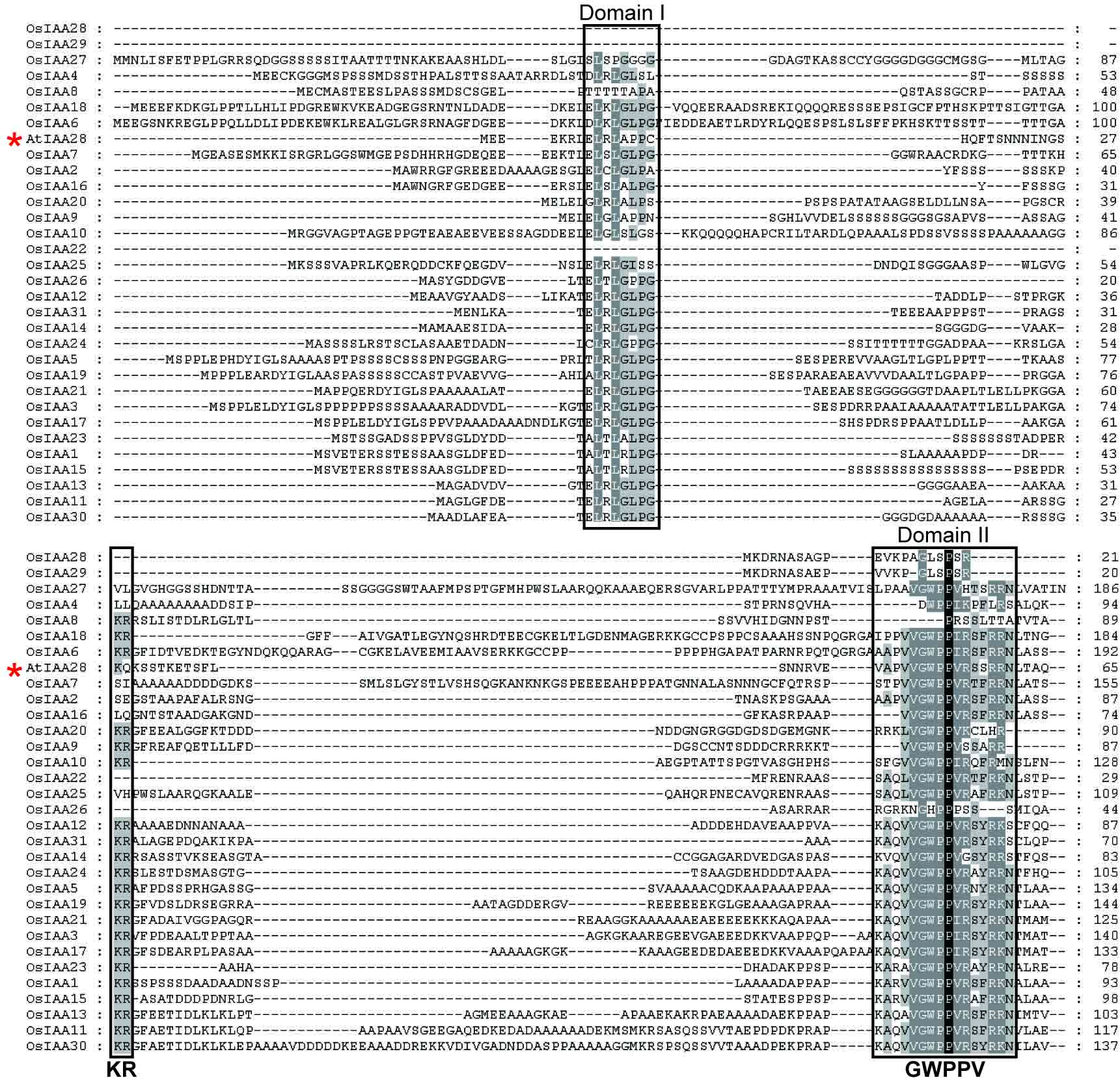
**

**Supplementary Figure S1 Sequence conservation between 31 OsAUX/IAAs and AtIAA28 proteins**

**Multiple alignments are fulfilled by MUSCLE 3.6 software. Only sequences, including Domain I and II, marked out by black squares, are analyzed. Asterisk represents IAA28 protein in *Arabidopsis*. Alignment of the** AUX/IAA **proteins showed that sequences of Domain I, II and KR** dipeptide are highly conserved between *Arabidopsis* and rice. This high level of conservation across species is possibly attributed to their functional importance during evolution. Domain I is responsible for its transcriptional repressor activity; Domain II is the motif interacting with auxin molecule and TIR1/AFBs, and determines the degron sequence GWPPV and the rapid degradation of Aux/IAA proteins required for auxin responsiveness; Dipeptide KR is a part of a putative bipartite nuclear localization sequence (NLS), and involved in guiding AUX/IAA proteins into cell nucleus.


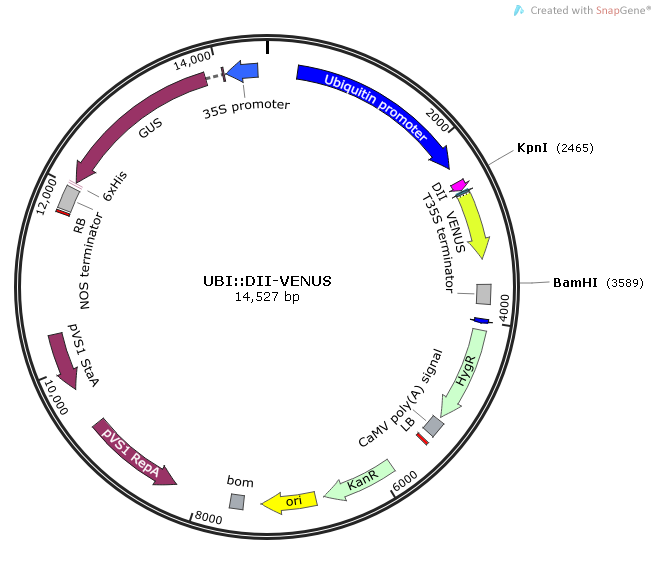


**Supplementary Figure S**2 **Schematic representation of UBI::DII-VENUS construct used for auxin response**

DII (KQ)-VENUS fragment was cloned from AtIAA28 coding sequence and then inserted under the maize ubiquitin-1 promoter using using Kpn I and Bam HI restriction sites. This backbone vector is pCAMBIA1301. HygR, hygromycin; KanR, kanamycin; LB, left border; RB, right border.**
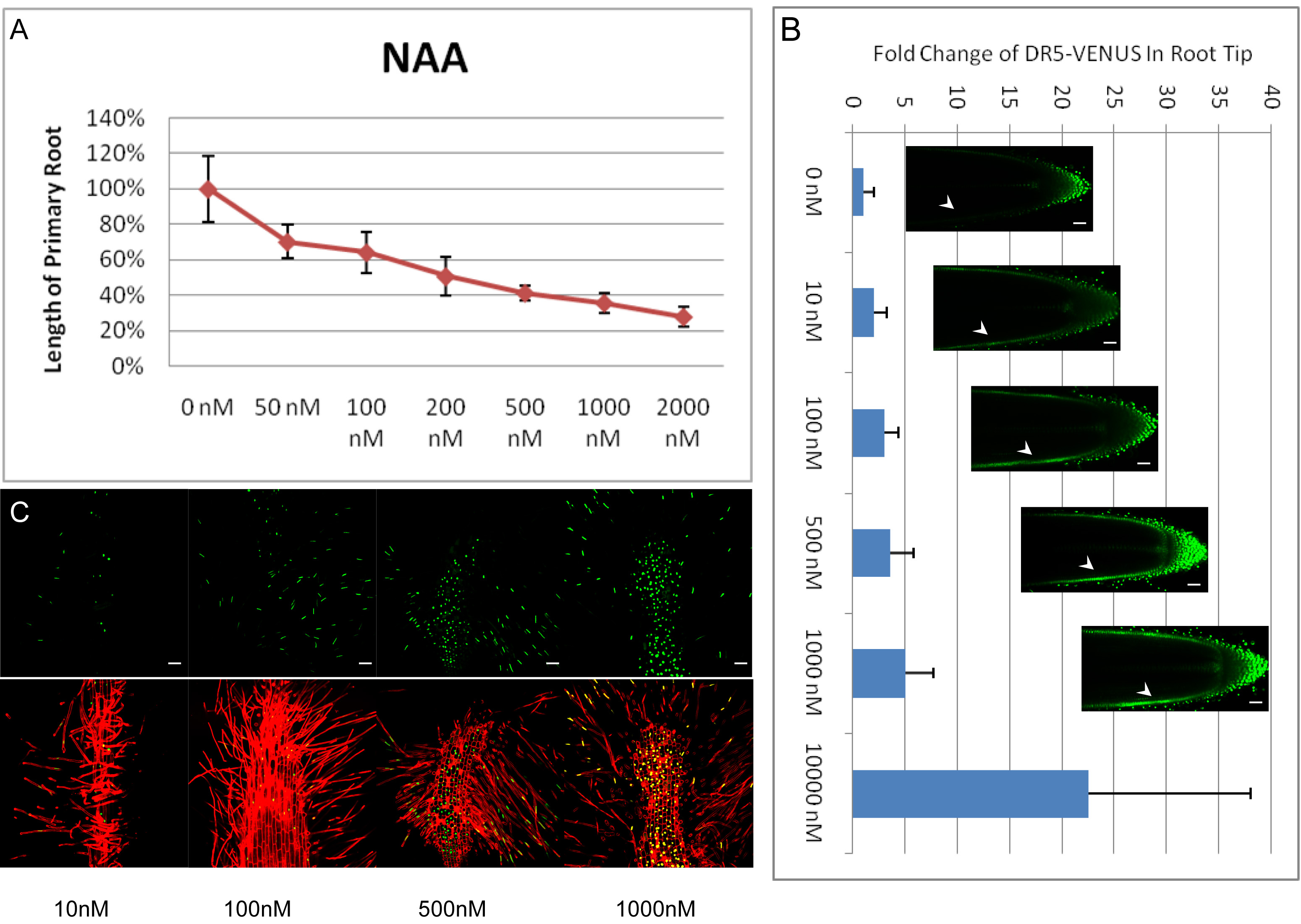
**

**Supplementary Figure S3 Behaviors of *DR5*-VENUS toward exogenous NAA application**

**3-days old *DR5*-VENUS seedlings treated with a rising NAA concentration.**

**(A) NAA inhibition of primary root elongation.** All the data were expressed as mean ±SEM **(Root Number≥10)**.

**(B) *DR5* signal measurements in rice root tip after NAA treatment. Intensity was evaluated using ImageJ.** All the data were expressed as mean ±SEM **(Root Number≥11).** Arrowheads marks the gradually increases of auxin signals at the epidermal layer site. **Scale bar:** 50μm.

**(C) *DR5* signal changes in root hair zone (root hair cells and epidermis) after NAA treatment. Red: PI, Green: VENUS. Scale bar:** 50μm.


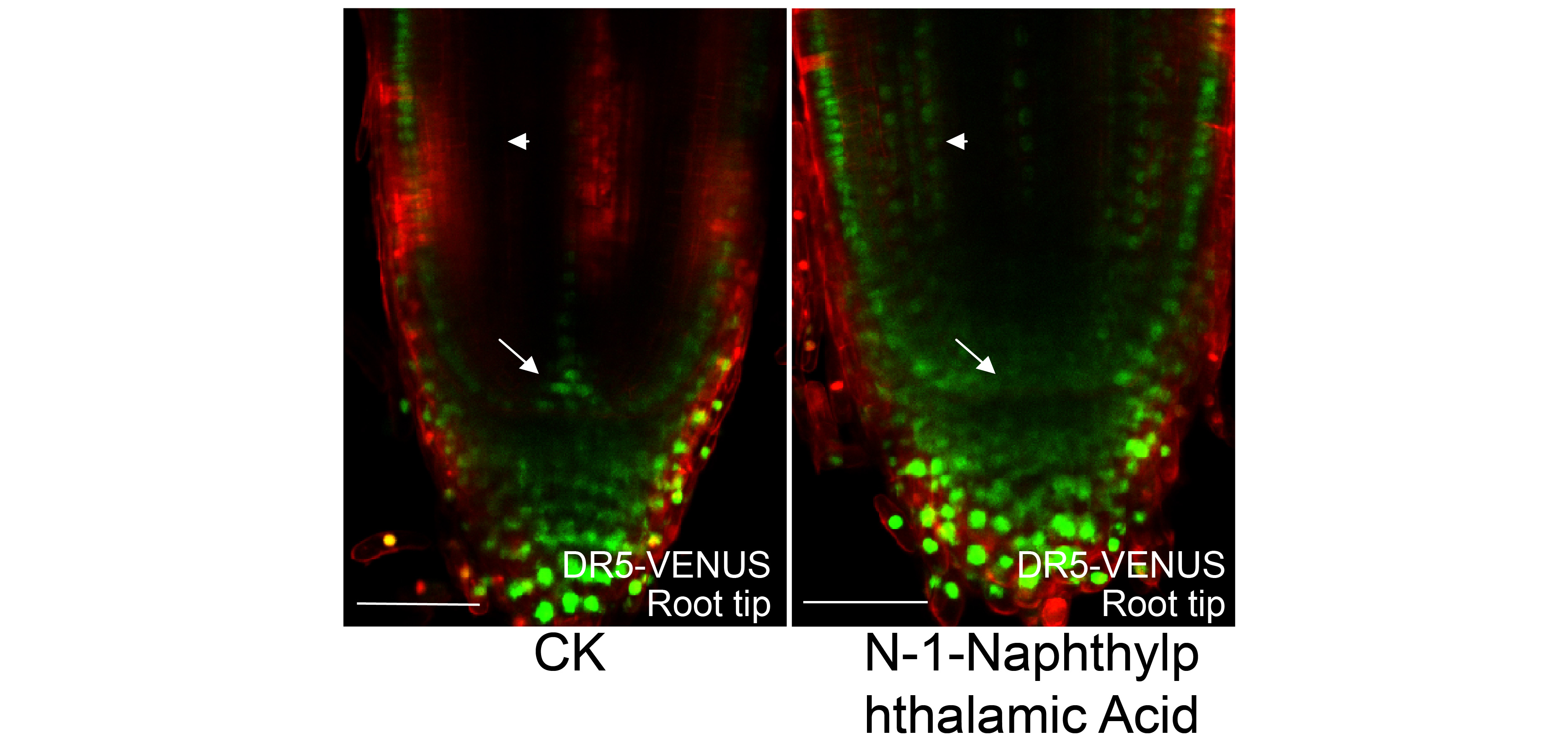


**Supplementary Figure S4 The magnified images of rice root tips for NPA treatment in Fig. 1C.**

Arrowheads indicates the ectopic expression of *DR5*-VENUS at cortex cells; Arrows marked out the *DR5*-VENUS pattern at root QC region. Red channel, propidium iodide; Green channel, VENUS. Scale bar: 50μm.

**
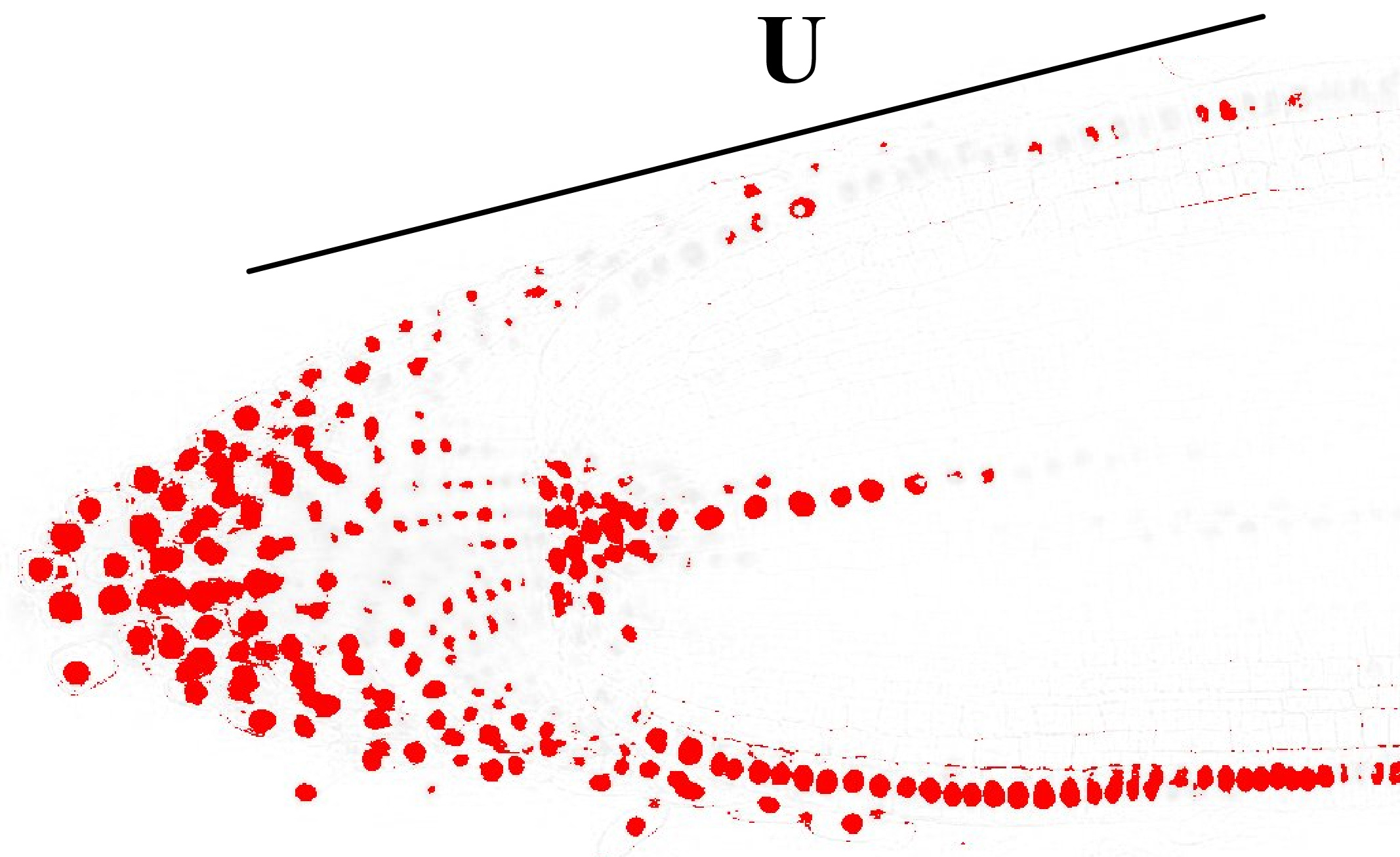
**
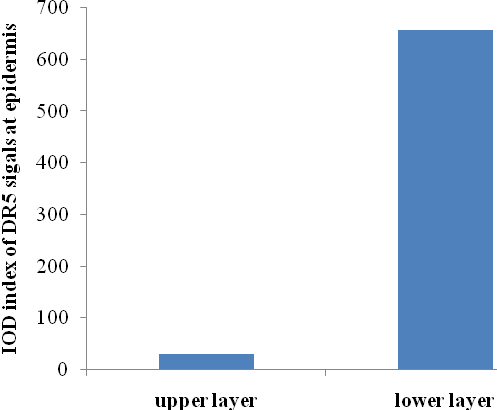


**Supplementary Figure S5 Quantification of *DR5*-VENUS fluorescence in the upper and lower domains in the gravistimulated root in Fig. 1F.**

Fluorescence was quantitatively analyzed using ImageJ software. We turned the image into bit-8 gray scale figure and inverted black and white color, then marked it using threshold settings (top panel), at last the total optical intensity (IOD) in the upper (marked by “U”) and lower epidermal layers were separately measured. Results (below panel) showed that *DR5-*-VENUS signals at the upper epidermis were largely attenuated. Red represents *DR5*-VENUS signals that can be caculated.


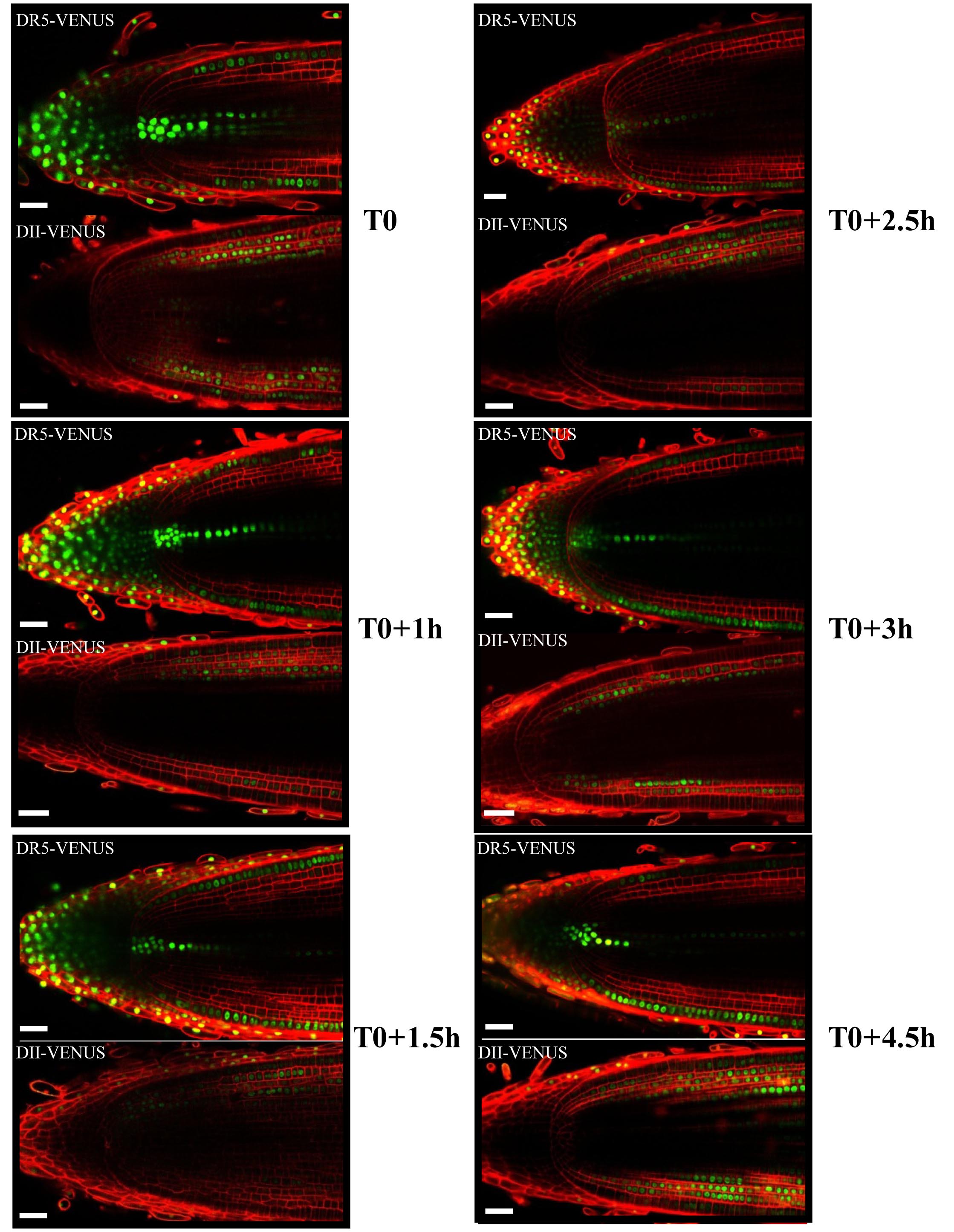
**Supplementary Figure S6 Gravitropism assays of rice *DR5*-VENUS and *DII-VENUS* root tips**

***DR5*-VENUS and *DII-VENUS* signals were followed during the 5 h gravitropism response. *DR5*-VENUS response at the upper epidermis layer was largely attenuated after 2.5 h** gravistimulation**. The most notable changes for *DII-VENUS* were observed at about 1.5 h after gravity stimulus. Images were taken using** LSM 7MP/OPO multiphoton microscope**. Scale bar: 25μm.**

**
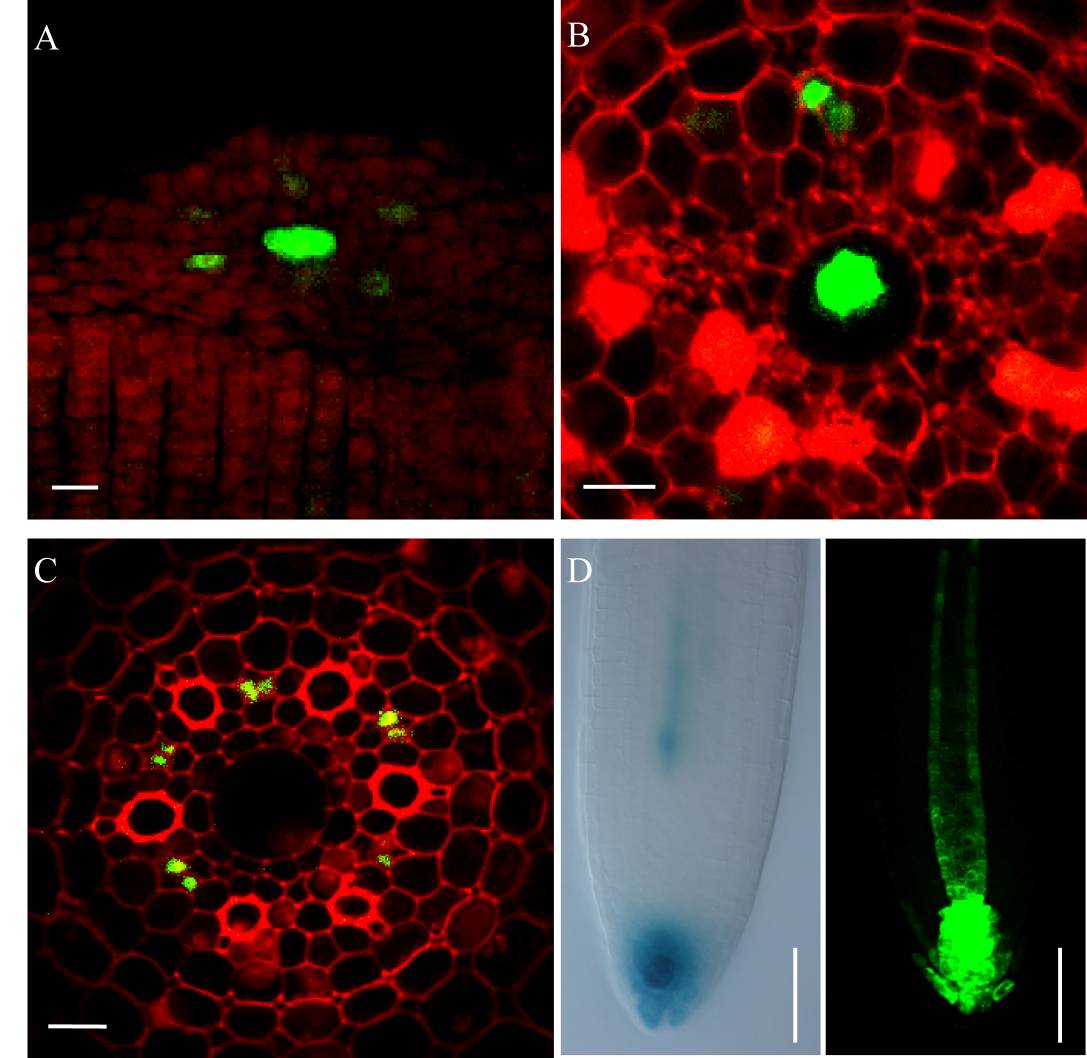
**

**Supplementary Figure S7 Auxin is mostly present in xylem and phloem cells in mature rice root vascular tissues**

**(A-C)** *DR5*-VENUS signal inside successive cross sections of radicle root tip at about 3 mm top of QC.

Auxin signals are observed inside metaxylem cells **(A)**, central metaxylem and newly-formed protoxylem cells **(B)**, and companion phloem cells **(C)**. Red channel, propidium iodide; Green channel, VENUS. Scale bar: 25μm.

**(D) *DR5*-GUS (Left) and *DR5*-GFP (Right) signals at protoxylem cell files in *Arabidopsis* root tip. Scale bar: 500μm.**

**
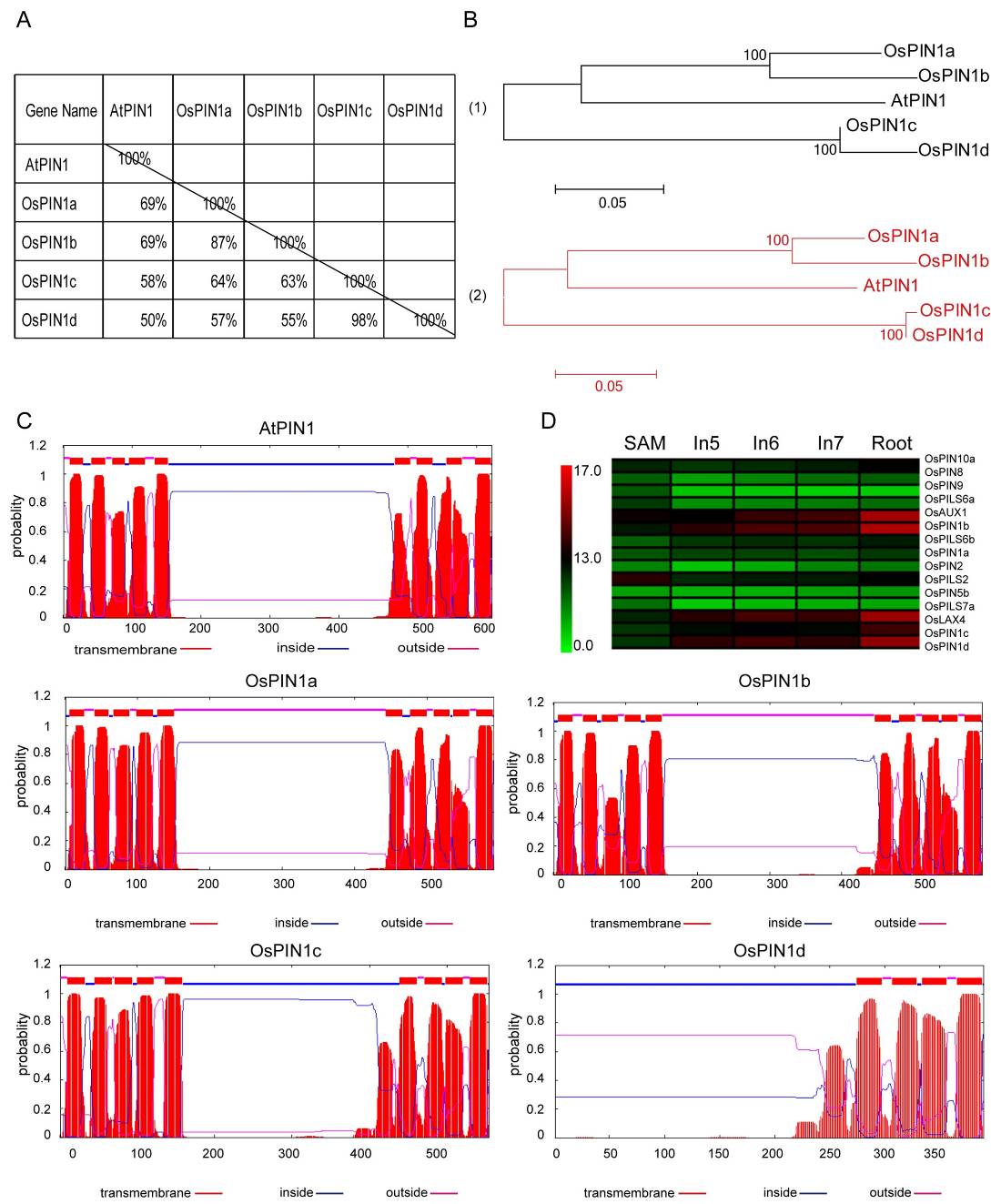
**

**Supplementary Figure S8 Gene and phylogenetic analysis of *OsPIN1s* and *OsAUX1* in Rice**

**(A)** Pairwise alignments between protein sequences of *OsPIN1s* and *AtPIN1* genes.

**(B)** OsPIN1s are closely related to AtPIN1. Phylogenic trees were generated by MEGA 3.0 with neighbor joining (NJ) (1) and Maximum Likelihood (ML) (2) algorithms.

**(C)** OsPIN1s proteins have similar topological structures with AtPIN1. Prediction of PIN1 protein transmembrane structures was carried out using TMHMM Server v. 2.0.

**(D)** Microarray data analysis of *OsPIN1s* and *OsAUX1* from four rice tissues. Red color represents the relative higher expression level, and green color represents the relative lower expression.

**
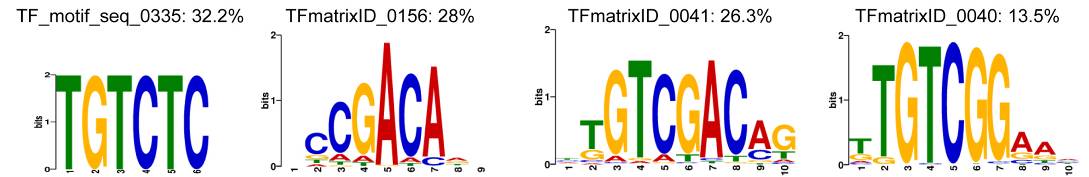
**

**Supplementary Figure S9 Four sequence logos of AuxRE motifs and their frequencies among rice GH3 gene family**

TF_motif_seq_0335 (TGTCTC) has the highest percentage among the four predicted motifs in 3000-bp upstream promoter regions of 11 OsGH3 members.

**Supplementary Table S1 Primer pairs used for quantitative RT-PCR analysis**

| **Primer** | **Sequence (5’ to 3’)** | **Gene Name**  (Locus Number) |
| --- | --- | --- |
| 1a-F | GGCATTGTCCCCTTCGTCTTCG | *OsPIN1a*  LOC_Os06g12610 |
| 1a-R | GCCCCAGCAGGATGTAGTACACCAG |
| 1b-2F | TGCACCCTAGCATTCTCAGCA | *OsPIN1b*  LOC_Os02g50960 |
| 1b-2R | CCCTCCTCCCAAATTCTACTTC |
| 1c-2F | TCGCACGGGACGCAGTCA | *OsPIN1c*  LOC_Os11g04190 |
| 1c-2R | CCCGTCCTTCTCGTTCTTGTTC |
| 1d-F | GGGAATTGAGATGCCGGCTA | *OsPIN1d*  LOC_Os12g04000 |
| 1d-R | CCAATGTGATGGGGAGAGCT |
| osAUX1-F | TCCTCGCCATCATCTTCCCCTT | *OsAUX1*  LOC_Os01g63770 |
| osAUX1-R | TGTTGAGCACGAACATCCCCGT |
| GH3.2-F | AGCCTTCTACTACAACTACTACT | *OsGH3.2*  LOC_Os01g55940 |
| GH3.2-R | TGACACTGACACCGACTG |
| RR3-F | CGCAGCTCCAAATATCGAGTTAC | *OsRR3*  LOC_Os02g58350 |
| RR3-R | CACATTCCGATCCAGGCTGAG |
| RR6-F | CACAATGTTGAAATGGCACCAG | *OsRR6*  LOC_Os04g57720 |
| RR6-R | AATTAATGTCAAAACTCTGACCGATC |
| tublin-F | GCTGACCACACCTAGCTTTGG | *Ostublinβ-4*  LOC_Os01g59150 |
| tublin-R | AGGGAACCTTAGGCAGCATGT |
| ubi-F | GAGCCTCTGTTCGTCAAGTA | *Ubiquitin 2*  LOC_Os02g06640 |
| ubi-R | ACTCGATGGTCCATTAAACC |

**Supplementary Table S2 List of potential ARSs in 11 OsGH3 family members and auxin responsive AtGH3.3 gene**

Targeted genes in red represent OsGH3.3, OsGH3.5, OsGH3.12 and OsGH3.10. OsGH3.3, OsGH3.5, OsGH3.12 contains the highest number of ARS sites in their promoter while OsGH3.10 had no ARS site. In *A. thaliana*, GH3.3 gene had only one ARS present in its 3000-bp promoter region .

| **Target Gene**  (Locus Number) | **Matrix ID** | **Family** | **Position** | **Strand** | **Similar Score** | **Sequence Logo-like** | **Direction** |
| --- | --- | --- | --- | --- | --- | --- | --- |
| OsGH3.13  LOC_Os11g32510 | TF_motif_seq_0335 | B3;ARF | -2227 | + | 1 | TGTCTc | forward |
|  | TF_motif_seq_0335 | B3;ARF | -2059 | - | 1 | TGTCTc | forward |
|  | TF_motif_seq_0335 | B3;ARF | -724 | - | 1 | TGTCTc | inverted |
|  | TF_motif_seq_0335 | B3;ARF | -1656 | - | 1 | TGTCTc | inverted |
|  | TF_motif_seq_0335 | B3;ARF | -284 | - | 1 | TGTCTc | inverted |
|  | TFmatrixID_0156 | B3;ARF | -1475 | - | 0.96 | ctTGTCGac | forward |
|  | TFmatrixID_0156 | B3;ARF | -2867 | + | 0.96 | gtTGTCGaa | inverted |
|  | TFmatrixID_0041 | B3;ARF | -1473 | + | 0.93 | tgGTCGAcaa | inverted |
|  | TFmatrixID_0041 | B3;ARF | -1473 | - | 0.93 | ttgTCGACca | forward |
|  | TFmatrixID_0041 | B3;ARF | -1668 | + | 0.87 | acGTCGAcga | inverted |
|  | TFmatrixID_0041 | B3;ARF | -1668 | - | 0.88 | tcgTCGACgt | forward |
|  | TFmatrixID_0041 | B3;ARF | -1935 | + | 0.84 | cgaTCGACag | inverted |
|  | TFmatrixID_0041 | B3;ARF | -1935 | - | 0.83 | cgaTCGACca | forward |
| OsGH3.12  LOC_Os11g08340 | TFmatrixID_0156 | B3;ARF | -1630 | + | 0.96 | atTGTCGag | forward |
|  | TFmatrixID_0156 | B3;ARF | -11 | + | 0.96 | atTGTCGca | forward |
|  | TF_motif_seq_0335 | B3;ARF | -2660 | + | 1 | TGTCTc | forward |
|  | TF_motif_seq_0335 | B3;ARF | -2646 | + | 1 | TGTCTc | forward |
|  | TF_motif_seq_0335 | B3;ARF | -1135 | + | 1 | TGTCTc | forward |
|  | TF_motif_seq_0335 | B3;ARF | -1105 | + | 1 | TGTCTc | forward |
|  | TF_motif_seq_0335 | B3;ARF | -181 | + | 1 | TGTCTc | forward |
|  | TF_motif_seq_0335 | B3;ARF | -1102 | + | 1 | TGTCTc | inverted |
| OsGH3.11  LOC_Os07g47490 | TF_motif_seq_0335 | B3;ARF | -2483 | - | 1 | TGTCTc | inverted |
|  | TF_motif_seq_0335 | B3;ARF | -1380 | + | 1 | TGTCTc | forward |
|  | TF_motif_seq_0335 | B3;ARF | -229 | - | 1 | TGTCTc | inverted |
|  | TFmatrixID_0156 | B3;ARF | -316 | - | 0.96 | ccTGTCGca | forward |
|  | TFmatrixID_0156 | B3;ARF | -2841 | + | 1 | ccTGTCGgt | inverted |
|  | TFmatrixID_0041 | B3;ARF | -1660 | + | 0.87 | taGTCGActc | inverted |
|  | TFmatrixID_0041 | B3;ARF | -1660 | - | 0.87 | gagTCGACta | forward |
|  | TFmatrixID_0041 | B3;ARF | -1699 | + | 0.88 | ccGTCGAcgt | inverted |
|  | TFmatrixID_0041 | B3;ARF | -1699 | - | 0.91 | acgTCGACgg | forward |
|  | TFmatrixID_0040 | B3;ARF | -2842 | + | 0.94 | ctGTCGGtcc | inverted |
| OsGH3.10  LOC_Os07g38860 | TFmatrixID_0156 | B3;ARF | -2024 | - | 0.96 | gaTGTCGccg | inverted |
|  | TFmatrixID_0156 | B3;ARF | -2641 | - | 0.96 | ctTGTCGAtg | forward |
| OsGH3.8  LOC_Os07g40290 | TF_motif_seq_0335 | B3;ARF | -516 | - | 1 | TGTCTc | inverted |
|  | TF_motif_seq_0335 | B3;ARF | -2324 | - | 1 | TGTCTc | forward |
|  | TFmatrixID_0156 | B3;ARF | -891 | - | 0.96 | ttTGTCGag | inverted |
|  | TFmatrixID_0156 | B3;ARF | -743 | - | 0.96 | ccTGTCGcc | inverted |
|  | TFmatrixID_0156 | B3;ARF | -280 | - | 1 | gcTGTCGgt | inverted |
|  | TFmatrixID_0156 | B3;ARF | -270 | - | 1 | gcTGTCGGt | forward |
|  | TFmatrixID_0141 | B3;ARF | -1390 | + | 0.89 | ccGTCGAcct | forward |
|  | TFmatrixID_0041 | B3;ARF | -1390 | - | 0.92 | aggTCGACgg | inverted |
|  | TFmatrixID_0040 | B3;ARF | -582 | - | 0.94 | gcTGTCGGtc | inverted |
|  | TFmatrixID_0040 | B3;ARF | -268 | - | 0.94 | cTGTCGGtga | forward |
| OsGH3.7  LOC_Os06g30440 | TFmatrixID_0040 | B3;ARF | -2579 | + | 0.95 | gtGTCGGgcc | forward |
|  | TFmatrixID_0041 | B3;ARF | -557 | - | 0.87 | ccGTCGACgc | inverted |
|  | TFmatrixID_0041 | B3;ARF | -557 | + | 0.91 | gcgTCGACgg | forward |
|  | TFmatrixID_0041 | B3;ARF | -151 | + | 0.89 | acGTCGAcct | inverted |
|  | TFmatrixID_0041 | B3;ARF | -151 | - | 0.89 | aggTCGACgt | forward |
|  | TFmatrixID_0156 | B3;ARF | -2580 | + | 0.99 | tgTGTCGgg | forward |
| OsGH3.5  LOC_Os05g50890 | TF_motif_seq_0335 | B3;ARF | -2726 | - | 1 | TGTCTc | inverted |
|  | TF_motif_seq_0335 | B3;ARF | -2451 | - | 1 | TGTCTc | inverted |
|  | TF_motif_seq_0335 | B3;ARF | -1829 | + | 1 | TGTCTc | forward |
|  | TF_motif_seq_0335 | B3;ARF | -1732 | + | 1 | TGTCTc | forward |
|  | TF_motif_seq_0335 | B3;ARF | -1282 | + | 1 | TGTCTc | forward |
|  | TF_motif_seq_0335 | B3;ARF | -1844 | + | 1 | TGTCTc | inverted |
|  | TFmatrixID_0156 | B3;ARF | -1409 | + | 0.96 | atTGTCGat | forward |
|  | TFmatrixID_0156 | B3;ARF | -1231 | + | 0.99 | tgTGTCGgt | forward |
|  | TFmatrixID_0156 | B3;ARF | -1108 | + | 0.96 | ttTGTCGag | forward |
|  | TFmatrixID_0156 | B3;ARF | -219 | + | 1 | tcTGTCGgc | forward |
|  | TFmatrixID_0156 | B3;ARF | -717 | + | 1 | ttTGTCGgt | inverted |
|  | TFmatrixID_0156 | B3;ARF | -1825 | + | 1 | tcTGTCGgt | inverted |
|  | TFmatrixID_0041 | B3;ARF | -1107 | + | 0.86 | ttGTCGAgag | forward |
|  | TFmatrixID_0040 | B3;ARF | -1230 | + | 0.95 | gtGTCGGtgt | forward |
|  | TFmatrixID_0040 | B3;ARF | -218 | + | 0.96 | ctGTCGGcaa | forward |
|  | TFmatrixID_0040 | B3;ARF | -717 | + | 0.97 | ttGTCGGtag | inverted |
|  | TFmatrixID_0040 | B3;ARF | -1825 | + | 0.95 | ctGTCGGtac | inverted |
| OsGH3.4  LOC_Os05g42150 | TF_motif_seq_0335 | B3;ARF | -85 | - | 1 | TGTCTc | inverted |
|  | TF_motif_seq_0335 | B3;ARF | -2144 | + | 1 | TGTCTc | inverted |
|  | TF_motif_seq_0335 | B3;ARF | -1568 | + | 1 | TGTCTc | inverted |
|  | TF_motif_seq_0335 | B3;ARF | -739 | - | 1 | TGTCTc | forward |
|  | TFmatrixID_0156 | B3;ARF | -981 | - | 1 | gtTGTCGgt | inverted |
|  | TFmatrixID_0156 | B3;ARF | -51 | + | 0.96 | acTGTCGct | inverted |
|  | TFmatrixID_0040 | B3;ARF | -983 | - | 0.96 | ttGTCGGttg | inverted |
| OsGH3.3  LOC_Os01g12160 | TF_motif_seq_0335 | B3;ARF | -373 | + | 1 | TGTCTc | inverted |
|  | TF_motif_seq_0335 | B3;ARF | -413 | + | 1 | TGTCTc | inverted |
|  | TF_motif_seq_0335 | B3;ARF | -2471 | - | 1 | TGTCTc | forward |
|  | TF_motif_seq_0335 | B3;ARF | -335 | + | 1 | TGTCTc | forward |
|  | TF_motif_seq_0335 | B3;ARF | -723 | + | 1 | TGTCTc | forward |
|  | TF_motif_seq_0335 | B3;ARF | -81 | + | 1 | TGTCTc | forward |
|  | TFmatrixID_0156 | B3;ARF | -207 | + | 1 | ttTGTCGgg | inverted |
|  | TFmatrixID_0156 | B3;ARF | -251 | + | 0.96 | gtTGTCGtc | inverted |
|  | TFmatrixID_0156 | B3;ARF | -271 | + | 0.96 | gtTGTCGat | inverted |
|  | TFmatrixID_0156 | B3;ARF | -730 | - | 0.96 | gtTGTCGaa | forward |
|  | TFmatrixID_0156 | B3;ARF | -1365 | - | 0.99 | ggTGTCGgg | forward |
|  | TFmatrixID_0156 | B3;ARF | -1431 | + | 0.96 | atTGTCGat | inverted |
|  | TFmatrixID_0040 | B3;ARF | -208 | + | 0.97 | ttGTCGGgca | inverted |
|  | TFmatrixID_0040 | B3;ARF | -1363 | - | 0.96 | gtGTCGGggt | forward |
|  | TFmatrixID_0040 | B3;ARF | -1863 | + | 0.87 | tgGTCGGaga | inverted |
|  | TFmatrixID_0041 | B3;ARF | -276 | + | 0.84 | ttGTCGAtcg | inverted |
|  | TFmatrixID_0041 | B3;ARF | -1432 | + | 0.84 | ttGTCGAtat | Inverted |
| OsGH3.2  LOC_Os01g55940 | TFmatrixID_0040 | B3;ARF | -1433 | - | 0.95 | gtTGTCGGcct | inverted |
|  | TFmatrixID_0041 | B3;ARF | -1671 | + | 0.84 | cgaTCGACag | forward |
|  | TFmatrixID_0041 | B3;ARF | -1200 | + | 0.91 | ttgTCGACtc | forward |
|  | TFmatrixID_0041 | B3;ARF | -1200 | - | 0.92 | gagTCGACaa | inverted |
|  | TFmatrixID_0041 | B3;ARF | -907 | - | 0.84 | cgaTCGACat | inverted |
|  | TFmatrixID_0041 | B3;ARF | -583 | - | 0.96 | gtgTCGACac | inverted |
|  | TFmatrixID_0041 | B3;ARF | -583 | + | 0.96 | gtGTCGAcac | forward |
|  | TFmatrixID_0041 | B3;ARF | -717 | + | 0.89 | ccGTCGAcct | inverted |
|  | TFmatrixID_0041 | B3;ARF | -717 | - | 0.92 | agGTCGAcgg | forward |
|  | TFmatrixID_0156 | B3;ARF | -1431 | - | 0.99 | cgTGTCGgc | inverted |
|  | TFmatrixID_0156 | B3;ARF | -1198 | - | 0.96 | ttTGTCGgc | inverted |
|  | TFmatrixID_0156 | B3;ARF | -1759 | - | 1 | aaTGTCGga | forward |
|  | TFmatrixID_0156 | B3;ARF | -2979 | + | 0.96 | ctTGTCGtc | inverted |
|  | TF_motif_seq_0335 | B3;ARF | -1394 | + | 1 | TGTCTc | forward |
|  | TFmatrixID_0040 | B3;ARF | -1757 | - | 0.98 | atGTCGGaat | forward |
| OsGH3.1  LOC_Os01g57610 | TFmatrixID_0040 | B3;ARF | -1974 | - | 0.99 | gtGTCGGaaa | forward |
|  | TFmatrixID_0041 | B3;ARF | -6 | + | 0.88 | acGTCGAcgt | inverted |
|  | TFmatrixID_0041 | B3;ARF | -6 | - | 0.88 | acgTCGACgt | forward |
|  | TFmatrixID_0041 | B3;ARF | -2170 | + | 0.88 | ggGTCGAcga | inverted |
|  | TFmatrixID_0041 | B3;ARF | -2170 | - | 0.88 | tcgTCGACcc | forward |
|  | TFmatrixID_0156 | B3;ARF | -1822 | - | 1 | ttTGTCGgt | forward |
|  | TFmatrixID_0156 | B3;ARF | -1977 | - | 0.99 | AGTGTCGga | forward |
|  | TF_motif_seq_0335 | B3;ARF | -242 | - | 1 | TGTCTc | forward |
|  | TF_motif_seq_0335 | B3;ARF | -263 | - | 1 | TGTCTc | forward |
|  | TF_motif_seq_0335 | B3;ARF | -371 | - | 1 | TGTCTc | forward |
|  | TF_motif_seq_0335 | B3;ARF | -1428 | + | 1 | TGTCTc | inverted |
|  | TF_motif_seq_0335 | B3;ARF | -1656 | - | 1 | TGTCTc | forward |
|  | TFmatrixID_0040 | B3;ARF | -1820 | - | 0.96 | ttGTCGGttt | forward |
| AtGH3.3  AT2G23170 | TFmatrixID_0156 | B3;ARF | -2857 | + | 1 | tcTGTCGga | forward |
|  | TFmatrixID_0040 | B3;ARF | -2876 | + | 0.97 | ctGTCGGatt | forward |
|  | TFmatrixID_0041 | B3;ARF | -139 | + | 0.92 | atGTCGAcgt | forward |
|  | TFmatrixID_0041 | B3;ARF | -139 | - | 0.92 | acgTCGACat | inverted |
|  | TF_motif_seq_0335 | B3;ARF | -176 | + | 1 | TGTCTc | forward |
